# PID Control for Uncertain Systems with Integral Measurements and DoS Attacks Using a Binary Encoding Scheme

**DOI:** 10.3390/e28020225

**Published:** 2026-02-15

**Authors:** Nan Hou, Yanshuo Wu, Hongyu Gao, Zhongrui Hu, Xianye Bu

**Affiliations:** 1 Sanya Offshore Oil & Gas Research Institute, Northeast Petroleum University, Sanya 572025, China; nanhou@nepu.edu.cn (N.H.);; 2 Artificial Intelligence Energy Research Institute, Northeast Petroleum University, Daqing 163318, China; 3 State Key Laboratory of Continental Shale Oil, Northeast Petroleum University, Daqing 163318, China; 4 Heilongjiang Provincial Key Laboratory of Networking and Intelligent Control, Northeast Petroleum University, Daqing 163318, China; 5 Research Center for Mathematics and Interdisciplinary Sciences, Northeast Petroleum University, Daqing 163318, China; 6 School of Electrical & Information Engineering, Northeast Petroleum University, Daqing 163318, China

**Keywords:** PID control, integral measurements, binary encoding scheme, DoS attack, parameter uncertainty

## Abstract

In this paper, an observer-based proportional-integral-derivative (PID) controller is designed for a class of uncertain nonlinear systems with integral measurements, denial-of-service (DoS) attacks and bounded stochastic noises under a binary encoding scheme (BES). Parameter uncertainty is involved with a norm-bounded multiplicative expression. Integral measurements are considered to reflect the delayed signal collection of sensor. For communication, BES is put into use in the signal transmission process from the sensor to the observer and from the controller to the actuator. Random bit flipping is described that may take place caused by channel noises, whose impact is described by a stochastic noise. Randomly occurring DoS attacks are taken account of that may appear due to the shared network, which block the transmitted signals totally. Three sets of Bernoulli-distributed random variables are adopted to reveal the random occurrence of uncertainties, bit flipping and DoS attacks. The aim of this paper is to design an observer-based PID controller which guarantees that the closed-loop system reaches exponential ultimate boundedness in mean square (EUBMS). By virtue of Lyapunov stability theory, stochastic analysis technique and matrix inequality method, a sufficient condition is developed for designing the observer-based PID controller such that the closed-loop system achieves EUBMS performance, and the ultimate upper bound of the controlled output is bounded and such a bound is minimized. The gain matrices of the observer-based controller are acquired explicitly by virtue of solving the solution to an optimized issue with several matrix inequality constraints. Two simulation examples are given which indicate the usefulness of the proposed control method in this paper adequately.

## 1. Introduction

The study of control issue has been prevailing due to its critical role in maintaining the stable and safe operation of systems in various areas such as industrial Internet, aerospace industry, and transportation industry [1,2,3,4,5]. For example, a controller is an indispensable part of the heating furnace, when the temperature deviates from (higher/lower than) the expected value, the designed controller would make a decision automatically to manage (turn down/up) the fuel input valve, then the heating furnace would keep at the expected temperature and incidents are avoided resulted from temperature deviations. Nowadays, proportional-integral-derivative (PID) control has been focused on by researchers as an effective control means, and different meaningful results have been published in the literature, see [6,7,8,9] and the references therein. The core target of PID control is to materialize fast response, stability and accurate control of system via adopting the cooperation of the proportional sector, the integral sector and the differential sector [10,11,12,13,14]. For instance, in [13], an observer-based PID control strategy has been proposed for systems with time-varying delays suffering from randomly occurring DoS or deception attacks, by which the following performance constraints have been achieved of the closed-loop system: (1) the exponentially mean-square input-to-state stability; and (2) ϱ-security in mean-square sense and the existence of un upper bound on the quadratic cost criterion.

Constrained phenomena usually exist in systems unavoidably, which include incomplete measurement information [15,16], noises [17,18,19], communication constraints [20,21], outliers [22] and bias [23], and a great deal of effort has been devoted to the analysis and synthesis problems for such systems in order to weaken the negative influence from these constrained phenomena on system performance. Note that most research have been executed depending on the basis that the measurement only relies on the state at the current time step. Actually, this is not always true considering the fact that the measurement signal may also involve states in the previous several time steps due to real-time signal processing and delayed data acquisition. Recently, as a type of constrained measurement phenomenon, the phenomenon of integral measurements has attracted researchers’ attention, which is constituted by the sum of states at the current time step and over a past time step interval [24,25,26,27,28]. For example, in [24], integral measurements have been concerned for multirate systems, and the distributed dynamic event-based recursive filtering issue has been settled using matrix Riccati equation method. In [27], the issues of state estimation and fault reconstruction have been tackled for discrete-time systems subject to integral measurements, and an unknown input observer has been designed which is capable of decoupling partial disturbances and attenuating the impact from the remaining undecouplable noises.

Nowadays, the digital communication is usually adopted as the signal transmission means in networked control systems owing to its advantages including strong anti-interference ability, easy encryption, convenient storage and processing, and high device integration [29,30,31,32,33]. In recent years, binary encoding scheme (BES) has been paid attention to with merits such as simple technique realization (with expressions “1” and “0”), easy conversion between binary and decimal data, and high reliability with binary data, by using which (1) the signal is quantized and encoded into binary bit strings (BBSs), (2) BBSs are transmitted through the binary symmetric channel (BSC), and (3) BBSs are received and decoded into the decimal data at the receiver side [34,35,36,37,38]. For example, in [34], recursive quantized Kalman filtering problem has been tackled based on BESs in the approximate minimum mean-square error sense. To be detailed, concerning the existence of communication link noises during the transmission process of BBSs, randomly occurring bit flipping has been taken into account which is reflected by bit-error rates. Nevertheless, a literature search reveals that the PID control issue with integral measurements has not been investigated adequately yet, needless to mention the situation that the BES is put into use, and this is one of the motivations of this paper to compensate this research absence.

With the convenience and advance brought by rapid development of networked systems and information technology, a side effect appears that communication using the shared Internet may be vulnerable to cyber attacks unavoidably launched by the malicious adversary [39,40,41]. Note that security has always been one of the most crucial aspects to be kept everywhere and at any time. Recently, more and more research results have been published about cyber attacks which are analyzed from viewpoints of defenders or attackers, both of which aim to establish protected and safe environments for peoples’ work and life [42,43]. Denial-of-service (DoS) attack is a common attack type, which destroys the information transmission sector and prohibits the receiver from acquiring any information in areas including petroleum industry and production manufacture. For assuring the normal working performance of systems under DoS attacks, security control has become a heated topic and the corresponding control issues have been settled [44,45]. In [44], stability has been guaranteed for systems under DoS attacks with a longer tolerable attack duration, and the relationship has been characterized between the frequency of DoS attacks and the upper limit of tolerable attack duration. Stochastic pulsing DoS attacks have been dealt with in [45], where the time between two consecutive attack instants obeys exponential distribution. An event-triggered control method has been designed which tolerates such attacks and maintains stability and Zeno-freeness of system by combining attack-active parts with attack-over parts.

As is well known, nonlinearity often exists in systems as a result of system complexity itself, component usage wastage and working condition changes. Ways to cope with nonlinearity include the elimination of nonlinearity via linearization (e.g., Taylor expansion, and using stochastic characteristics) and the introduction of inequality constraints (Lipschitz condition, and sector-bounded condition). Control for nonlinear systems has attracted researchers’ interest, and a series of control methods has been developed which helps systems resist nonlinearity effectively [46,47,48]. Uncertainties exist in systems unneglectably owing to modelling error and changeable environment situations. In order to mitigate the impact from uncertainties on the system operation, research work has been done with respect to uncertain systems [49,50,51,52]. Taking [49] as an example, unmatched uncertainties have been taken into account when discussing prescribed-instant stability for second-order systems, and such a system has been propelled to the origin definitely at the required time step. Usually, noises appear in systems and affect on system behaviors which include the stochastic type, the bounded type, and the attenuated type [53,54,55,56]. In spite of its obvious research significance, the security control issue against DoS attacks with integral measurements utilizing BES has not been paid enough attention due possibly to its complexity, let alone the case that stochastic nonlinearities, uncertainty and bounded stochastic noises are taken account of simultaneously, which constitutes another motivation of this paper.

In summary, an observer-based PID controller is designed in this paper for uncertain nonlinear systems with integral measurements and DoS attacks using a BES. Difficulties to be settled involve (1) how to formulate this security-guaranteed observer-based PID control issue, utilizing a BES, mathematically and entirely within an unified framework? (2) how to handle the influence from randomly occurring bit flipping and DoS attacks on the controller performance? and (3) how to design the gains of the desired observer-based PID controller by which the closed-system realizes exponential ultimate boundedness in mean square (EUBMS)? Contributions of this paper are emphasized below: (1) a novel observer-based PID control approach is firstly put forth for a kind of uncertain nonlinear systems suffering from integral measurements and DoS attacks using a BES; (2) a sufficient condition is put forward such that the closed-loop system attains EUBMS performance, and the controlled output signal has a minimized upper bound of its ultimate upper bound; and (3) gain parameters of the controller and the observer are yielded directly via solving the solution to an optimized issue minimizing the upper bound of the ultimate upper bound of the controlled output restrained by several matrix inequalities.

The structure of this paper is organized as follows. In Section 1, the engineering background and main contents of this paper are stated, respectively. In Section 2, the mathematical models including the system and the observer-based controller are constructed and the problem to be settled is clarified. In Section 3, the EUBMS performance is analyzed for the closed-loop system and the control approach is yielded. In Section 4, two simulation examples are conducted to testify the effectiveness of the developed control approach. In Section 5, conclusions are yielded by summarizing the results attained in this paper.

**Notation** **1.** *Symbols in this paper is general except explained in particular. $R^{m}$ stands for m-dimensional Euclidean space where m is an arbitrary positive integer. For an arbitrary matrix Y, $Y^{T}$ and $Y^{⊥}$ show the transpose and the orthogonal basis for the null space, respectively. For an arbitrary symmetric matrix A, A>0 (or A<0) and A≥0 (or A≤0) mean that A is positive (or negative) definite and positive (or negative) semi-definite. For an arbitrary square matrix Q, tr(Q), $\lambda_{\min}\left(Q\right)$ (or $\lambda_{\max}\left(Q\right)$) and $Q^{-1}$ represent the trace, the minimum (or maximum) eigenvalue and the inverse of Q, respectively. diag{·} is an arbitrary block diagonal matrix. E{a} and V{a} express the expectation and the variance of an arbitrary random variable (or vector) a. Prob{·} depicts the occurrence probability of an event “·”. $I_{\left(l\right)}$ is an l×l identity matrix. ∥v∥ expresses the norm of an arbitrary vector v.*

## 2. Problem Formulation and Preliminaries

### 2.1. The System Model

In view of a class of uncertain nonlinear systems as follows:

(1) $$\begin{array}{cc} x(k+1)= & \left(A+\alpha (k)\Delta A)x(k)+Bu(k)+f(k,x(k))+Gw(k\right)\\ z(k)= & Hx(k) \end{array}$$

where $x\left(k\right)\in R^{n_{x}}$, $u\left(k\right)\in R^{n_{u}}$ and $z\left(k\right)\in R^{n_{z}}$ mean the system state vector, the control input vector and the controlled output vector, respectively. $w\left(k\right)\in R^{n_{w}}$ is the bounded stochastic process noise with E{w(k)}=0 and $V\left\{w\left(k\right)\right\}=w_{0}I>0$. *A*, *B*, *G* and *H* are given system parameters.

ΔA is the parameter uncertainty with the following form:

$$\begin{array}{c} \Delta A=EFN \end{array}$$

where *E* and *N* are known matrices, and *F* is un unknown matrix satisfying $F^{T}F\leq I$.

α(k)∈R is a Bernoulli-distributed random variable reflecting the random occurrence of parameter uncertainty, whose probability is as follows:

$$\begin{array}{cc} \; & \mathrm{Prob}\left\{\alpha \left(k\right)=1\right\}=\bar{\alpha },\mathrm{Prob}\left\{\alpha \left(k\right)=0\right\}=1-\bar{\alpha } \end{array}$$

where $\bar{\alpha }\in \left[0,1\right]$ is a known scalar.

f(k,x(k)) is a stochastic nonlinear function whose property is expressed below:

(2) $$\begin{array}{cc} \; & E\{f(k,x(k))|x(k)\}=0,\\ \; & E\{f\left(k,x\left(k\right)\right)f^{T}\left(j,x\left(j\right)\right)|x\left(k\right)\}=0,\;k\neq j,\\ \; & E\left\{f\left(k,x\left(k\right)\right)f^{T}\left(k,x\left(k\right)\right)|x\left(k\right)\right\}=\sum_{l=1}^{\xi }\iota_{l}\iota_{l}^{T}E\left\{x^{T}\left(k\right)\Gamma_{l}x\left(k\right)\right\}=\sum_{l=1}^{\xi }\Theta_{l}E\left\{x^{T}\left(k\right)\Gamma_{l}x\left(k\right)\right\} \end{array}$$

where $\Theta_{l}$ and $\Gamma_{l}$ (l=1,2,…,ξ) are known matrices, and ξ≥1 is a known scalar.

### 2.2. The Measurement Model

#### 2.2.1. The Integral Measurements

Noticing that delays may exist during the process of measurement data collection, the measurement signal at the current time step may contain both the current system state and system states in the previous certain time steps. This phenomenon is described by the integral measurements model below:

(3) $$\begin{array}{c} y\left(k\right)=C\sum_{\gamma =0}^{s}x\left(k-\gamma \right)+Dv\left(k\right) \end{array}$$

where $y\left(k\right)\in R^{n_{y}}$ is the measurement output, and $v\left(k\right)\in R^{n_{v}}$ is the bounded stochastic measurement noise with E{v(k)}=0 and $V\left\{v\left(k\right)\right\}=v_{0}I>0$. s>0 is a scalar indicating the previous time step length. *C* and *D* are given parameters.

Denoting $\eta \left(k\right)≜{\left[\begin{array}{cccc} x^{T}\left(k\right) & x^{T}\left(k-1\right) & \cdots & x^{T}\left(k-s\right) \end{array}\right]}^{T}$, one yields from (1) and (3) that

(4) $$\begin{array}{cc} \eta (k+1)= & \bar{A}\eta \left(k\right)+\alpha \left(k\right)\Delta \bar{A_{1}}\eta \left(k\right)+\bar{B}u\left(k\right)+I_{f}f\left(k,x\left(k\right)\right)+\bar{G}w\left(k\right),\\ y(k)= & \bar{C}\eta \left(k\right)+Dv\left(k\right),\\ z(k)= & \bar{H}\eta \left(k\right) \end{array}$$

where

$$\begin{array}{cc} \; & \bar{A}≜\underset{s+1}{\left[\underset{︸}{\begin{array}{ccccc} A & 0 & 0 & \cdots & 0\\ I & 0 & 0 & \cdots & 0\\ 0 & I & 0 & \cdots & 0\\ \vdots & \vdots & \ddots & \ddots & \vdots \\ 0 & 0 & \cdots & I & 0 \end{array}}\right]},\bar{G}≜{\left[G^{T}\;\;\underset{s}{\underset{︸}{\begin{array}{cccc} 0 & 0 & \cdots & 0 \end{array}}}\right]}^{T},\bar{B}≜{\left[B^{T}\;\;\underset{s}{\underset{︸}{\begin{array}{cccc} 0 & 0 & \cdots & 0 \end{array}}}\right]}^{T},\\ \; & I_{f}≜{\left[I\;\;\underset{s}{\underset{︸}{\begin{array}{cccc} 0 & 0 & \cdots & 0 \end{array}}}\right]}^{T},\Delta \bar{A_{1}}≜\mathrm{diag}\left\{\Delta A,\underset{s}{\underset{︸}{0,\ldots ,0}}\right\},\bar{C}≜\left[\underset{s+1}{\underset{︸}{\begin{array}{cccc} C & C & \cdots & C \end{array}}}\right],\\ \; & \bar{H}≜\left[H\;\;\underset{s}{\underset{︸}{\begin{array}{ccc} 0 & \cdots & 0 \end{array}}}\right]. \end{array}$$

#### 2.2.2. The BES

In this part, BES is adopted for signal transmission both from sensor to observer and from controller to actuator [38]. Considering the fact that only a finite bit budget of network channel is used for signal encoding due to the restricted communication bandwidth, quantization is necessary to pretreat signals. For signals y(k) and $\bar{u}\left(k\right)$ in this paper to be transmitted, a finite-length BBS is obtained through quantization and encoding, which is then sent over a memoryless BSC.

Presume that the size of a scalar signal ℏ(k) (the *ℓ*th element of y(k) with *ℓ* $=1,2,\ldots ,n_{y}$, or the ℵth element of $\bar{u}\left(k\right)$ with $ℵ=1,2,\ldots ,n_{u}$) is within a range [−ℑ,ℑ], where ℑ∈R>0 is application-dependent. A BBS with length ð is obtained by a binary encoder from encoding ℏ(k). The range [−ℑ,ℑ] is split into $2^{ð}-1$ portions with a uniform interval length shown below:

(5) $$\begin{array}{c} \theta =2ℑ/(2^{ð}-1). \end{array}$$

Signify the $2^{ð}$ points (both endpoints and points inside) which are spaced uniformly as

$$\begin{array}{c} O≜\{\kappa^{\left[1\right]},\kappa^{\left[2\right]},\kappa^{\left[3\right]},\ldots ,\kappa^{\left[2^{ð}\right]}\} \end{array}$$

where $\kappa^{\left[a\right]}≜-ℑ+\left(a-1\right)\theta$ with $a=1,2,\ldots ,2^{ð}$.

The following stochastic quantization function Q(k) is chosen to quantize the signal ℏ(k):

(6) $$\begin{array}{c} Q(k):\hbar (k)\to q(k,\hbar (k),ð) \end{array}$$

where q(k,ℏ(k),ð) represents the quantized signal. For symbol simplification, we define $\bar{\hbar }\left(k\right)≜q\left(k,\hbar \left(k\right),ð\right)$. When $\kappa^{\left[a\right]}\leq \hbar \left(k\right)\leq \kappa^{\left[a+1\right]}$, the quantized signal $\bar{\hbar }\left(k\right)$ is determined according to the probabilistic way below:

(7) $$\begin{array}{cc} \; & \mathrm{Prob}\left\{\bar{\hbar }\left(k\right)=\kappa^{\left[a\right]}\right\}=1-{\bar{q}}_{\hbar }\left(k\right),\mathrm{Prob}\left\{\bar{\hbar }\left(k\right)=\kappa^{\left[a+1\right]}\right\}={\bar{q}}_{\hbar }\left(k\right) \end{array}$$

where ${\bar{q}}_{\hbar }\left(k\right)≜\frac{\hbar \left(k\right)-\kappa^{\left[a\right]}}{\theta }$ and $0\leq {\bar{q}}_{\hbar }\left(k\right)\leq 1$. In particular, we simply get the probability of taking value of $\bar{\hbar }\left(k\right)$, which depends on the size of ℏ(k).

Define

(8) $$\begin{array}{c} {\tilde{q}}_{\hbar }\left(k\right)≜\bar{\hbar }\left(k\right)-\hbar \left(k\right) \end{array}$$

as the quantization error. Viewing (7) and (8), one recognizes that ${\tilde{q}}_{\hbar }\left(k\right)$ belongs to a Bernoulli-distribution stochastic variable with a probability below:

(9) $$\begin{array}{c} \mathrm{Prob}\left\{{\tilde{q}}_{\hbar }\left(k\right)=-{\bar{q}}_{\hbar }\left(k\right)\theta \right\}=1-{\bar{q}}_{\hbar }\left(k\right),\mathrm{Prob}\left\{{\tilde{q}}_{\hbar }\left(k\right)=\left(1-{\bar{q}}_{\hbar }\left(k\right)\right)\theta \right\}={\bar{q}}_{\hbar }\left(k\right). \end{array}$$

From (9), the following stochastic property is yielded:

(10) $$\begin{array}{c} E\left\{{\tilde{q}}_{\hbar }\left(k\right)\right\}=0,V\left\{{\tilde{q}}_{\hbar }\left(k\right)\right\}={\overset{ˇ}{q}}_{\hbar }\left(k\right)\leq \theta^{2}/4 \end{array}$$

where ${\overset{ˇ}{q}}_{\hbar }\left(k\right)≜\theta^{2}{\bar{q}}_{\hbar }\left(k\right)\left(1-{\bar{q}}_{\hbar }\left(k\right)\right)$. As quantization is conducted separately for ℏ(k), one gets that ${\tilde{q}}_{\hbar }\left(k\right)$ display mutual independence.

In order to transform the quantized signal $\bar{\hbar }\left(k\right)$ into binary bits, an encoding function M(k) is employed below:

(11) $$\begin{array}{c} M\left(k\right):\bar{\hbar }\left(k\right)\to B\left(k\right) \end{array}$$

where $B\left(k\right)≜\{{♭}^{\left(1\right)}\left(k\right),{♭}^{\left(2\right)}\left(k\right),\ldots ,{♭}^{\left(ð\right)}\left(k\right)\}$ (${♭}^{\left(b\right)}\left(k\right)\in \left\{0,1\right\},b=1,2,\ldots ,ð$) signifies the BBS acquiring via

(12) $$\begin{array}{c} \bar{\hbar }\left(k\right)=-ℑ+\sum_{b=1}^{ð}{♭}^{\left(b\right)}\left(k\right)2^{b-1}\theta . \end{array}$$

After encoding, BBS B(k) is sent over a memoryless BSC, where each bit may flip according to a probability (called crossover probability) resulted from channel noises. The received BBS is expressed below:

$$\begin{array}{cc} D(k)≜ & \left\{\varsigma^{\left(1\right)}\left(k\right),\varsigma^{\left(2\right)}\left(k\right),\ldots ,\varsigma^{\left(ð\right)}\left(k\right)\right\},\varsigma^{\left(b\right)}\left(k\right)\in \left\{0,1\right\},b=1,2,\ldots ,ð \end{array}$$

where $\varsigma^{\left(b\right)}\left(k\right)={϶}^{\left(b\right)}\left(k\right)\left(1-{♭}^{\left(b\right)}\left(k\right)\right)+\left(1-{϶}^{\left(b\right)}\left(k\right)\right){♭}^{\left(b\right)}\left(k\right)$ with

(13) $$\begin{array}{c} {϶}^{\left(b\right)}\left(k\right)=\left\{\begin{array}{c} 1,\;\mathrm{bit}\mathrm{flips}\mathrm{in}\mathrm{the}b\mathrm{th}\mathrm{bit}\\ 0,\;\mathrm{bit}\mathrm{does}\mathrm{not}\mathrm{flip}\mathrm{in}\mathrm{the}b\mathrm{th}\mathrm{bit}. \end{array}\right. \end{array}$$

${϶}^{\left(b\right)}\left(k\right)$ is with the probability below:

$$\begin{array}{cc} \; & \mathrm{Prob}\left\{{϶}^{\left(b\right)}\left(k\right)=1\right\}=\bar{϶},\mathrm{Prob}\left\{{϶}^{\left(b\right)}\left(k\right)=0\right\}=1-\bar{϶} \end{array}$$

where $\bar{϶}\in \left[0,1\right]$ means the crossover probability.

For promoting subsequent discussion, an assumption is utilized below.

**Assumption** **1.** *In (13), ${϶}^{\left(b\right)}\left(k\right)$(b=1,2,…,ð) are independent mutually and distributed identically.*

For decoding the received BBS D(k), a decoding function T(k) is used below:

(14) $$\begin{array}{c} T\left(k\right):D\left(k\right)\to q^{o}\left(k,\hbar \left(k\right),ð\right) \end{array}$$

where $q^{o}\left(k,\hbar \left(k\right),ð\right)$ characterizes the restored signal received after channel transmission with an expression below:

(15) $$\begin{array}{c} q^{o}\left(k,\hbar \left(k\right),ð\right)=-ℑ+\sum_{b=1}^{ð}\varsigma^{\left(b\right)}\left(k\right)2^{b-1}\theta . \end{array}$$

Set $\overset{˘}{\hbar }\left(k\right)≜q^{o}\left(k,\hbar \left(k\right),ð\right)$. Notice that $\varsigma^{\left(b\right)}\left(k\right)$ (b=1,2,…,ð) are independent mutually (as well as different $\overset{˘}{\hbar }\left(k\right)$). Lemma 1 is given below for subsequent utilization.

**Lemma** **1** **([**38**]).** *For a signal $\bar{\hbar }\left(k\right)$ transmitted over a memoryless BSC with a crossover probability $\bar{϶}$, the expectation and the variance of the received signal $\overset{˘}{\hbar }\left(k\right)$ are manifested below:*

(16) $$\begin{array}{c} E\left\{\overset{˘}{\hbar }\left(k\right)\right\}=\left(1-2\bar{϶}\right)\bar{\hbar }\left(k\right),\;V\left\{\overset{˘}{\hbar }\left(k\right)\right\}={ℑ}^{2}\pi \end{array}$$

*where $\pi ≜\frac{4\bar{϶}\left(1-\bar{϶}\right)\left(2^{2ð}-1\right)}{3{\left(2^{ð}-1\right)}^{2}}$ and expectation is taken about the random variables ${϶}^{\left(b\right)}\left(k\right)$.*

Taking account of (16), one gets that the signals received $\overset{˘}{\hbar }\left(k\right)$ deviate from the quantized signals $\bar{\hbar }\left(k\right)$. In order to remove such distortions, we signify

(17) $$\begin{array}{c} \tilde{\hbar }\left(k\right)≜\frac{1}{1-2\bar{϶}}\overset{˘}{\hbar }\left(k\right) \end{array}$$

as the recovered signal. From (16) and (17), we recognize that $E\left\{\tilde{\hbar }\left(k\right)\right\}=\bar{\hbar }\left(k\right)$. Set

(18) $$\begin{array}{c} {\tilde{r}}_{\hbar }\left(k\right)≜\tilde{\hbar }\left(k\right)-\bar{\hbar }\left(k\right) \end{array}$$

as the noise describing impact from random bit errors. In terms of (16)–(18), one has

(19) $$\begin{array}{c} E\left\{{\tilde{r}}_{\hbar }\left(k\right)\right\}=0,V\left\{{\tilde{r}}_{\hbar }\left(k\right)\right\}=\left(1/{\left(1-2\bar{϶}\right)}^{2}\right){ℑ}^{2}\pi . \end{array}$$

Combining (8) with (18), the recovered measurement is described below:

(20) $$\begin{array}{c} \tilde{\hbar }\left(k\right)=\hbar \left(k\right)+{\tilde{q}}_{\hbar }\left(k\right)+{\tilde{r}}_{\hbar }\left(k\right). \end{array}$$

#### 2.2.3. The Randomly Occurring DoS Attacks

Replacing ℏ(k) with measurement signal component $y_{\ell }\left(k\right)$ in (20), we have

(21) $$\begin{array}{c} {\tilde{y}}_{\ell }\left(k\right)=y_{\ell }\left(k\right)+{\tilde{q}}_{y_{\ell }}\left(k\right)+{\tilde{r}}_{y_{\ell }}\left(k\right),\ell =1,2,\ldots ,n_{y}. \end{array}$$

Define

$$\begin{array}{cc} ϝ(k)≜ & {\left[\begin{array}{cccc} {ϝ}_{1}\left(k\right) & {ϝ}_{2}\left(k\right) & \cdots \; & {ϝ}_{n_{y}}\left(k\right) \end{array}\right]}^{T}\left(ϝ=y,\bar{y},{\tilde{q}}_{y},\overset{˘}{y},\tilde{y},{\tilde{r}}_{y}\right). \end{array}$$

Viewing (10) and (19), the statistical properties of ${\tilde{q}}_{y}\left(k\right)$ and ${\tilde{r}}_{y}\left(k\right)$ are expressed as follows:

(22) $$\begin{array}{cc} \; & E\left\{{\tilde{q}}_{y}\left(k\right)\right\}=0,V\left\{{\tilde{q}}_{y}\left(k\right)\right\}\leq \theta^{2}/4I_{\left(n_{y}\right)},\\ \; & E\left\{{\tilde{r}}_{y}\left(k\right)\right\}=0,V\left\{{\tilde{r}}_{y}\left(k\right)\right\}=\left(1/{\left(1-2\bar{϶}\right)}^{2}\right){ℑ}^{2}\pi I_{\left(n_{y}\right)}. \end{array}$$

Noticing that DoS attacks may occur during the network transmission process, one expresses the actually received measurement signal as follows:

(23) $$\begin{array}{c} \overset{`}{y}\left(k\right)=\left(1-\beta_{1}\left(k\right)\right)\tilde{y}\left(k\right) \end{array}$$

where $\beta_{1}\left(k\right)$ is a random variable indicating the random occurrence of DoS attacks, whose distribution is shown below:

(24) $$\begin{array}{c} \mathrm{Prob}\left\{\beta_{1}\left(k\right)=1\right\}=\bar{\beta },\mathrm{Prob}\left\{\beta_{1}\left(k\right)=0\right\}=1-\bar{\beta } \end{array}$$

with a scalar $\bar{\beta }\in \left[0,1\right]$. We see from (23) that when $\beta_{1}\left(k\right)=1$, DoS attacks occur, and we get $\overset{`}{y}\left(k\right)=0$; when $\beta_{1}\left(k\right)=0$, DoS attacks do not occur, and we have $\overset{`}{y}\left(k\right)=\tilde{y}\left(k\right)$.

### 2.3. The Observer-Based PID Controller Model

For system (4), based on the received measurement $\overset{`}{y}\left(k\right)$, the following observer-based PID controller is constructed:

(25) $$\begin{array}{cc} \; & \hat{x}\left(k+1\right)=\bar{A}\hat{x}\left(k\right)+L(\overset{`}{y}\left(k\right)-\bar{C}\hat{x}\left(k\right))+\bar{B}u\left(k\right),\\ \; & \bar{u}\left(k\right)=K_{P}\hat{x}\left(k\right)+K_{I}\sum_{l=k-N}^{k-1}\hat{x}\left(l\right)+K_{D}\left(\hat{x}\left(k\right)-\hat{x}\left(k-1\right)\right) \end{array}$$

where $\hat{x}\left(k\right)\in R^{n_{x}\times \left(s+1\right)}$ represents the estimate of η(k), $\bar{u}\left(k\right)\in R^{n_{u}}$ is the controller output signal, *L*, $K_{P}$, $K_{I}$ and $K_{D}$ are unknown gains to be decided. *N* is a given scalar indicating time length.

During the transmission of ${\bar{u}}_{ℵ}\left(k\right)$ ($ℵ=1,2,\ldots ,n_{u}$) from the controller to the actuator, the BES is employed. Substituting ℏ(k) in (20) with ${\bar{u}}_{ℵ}\left(k\right)$, one attains

(26) $$\begin{array}{c} {\tilde{\bar{u}}}_{ℵ}\left(k\right)={\bar{u}}_{ℵ}\left(k\right)+{\tilde{q}}_{{\bar{u}}_{ℵ}}\left(k\right)+{\tilde{r}}_{{\bar{u}}_{ℵ}}\left(k\right). \end{array}$$

Set

$$\begin{array}{cc} ℷ(k)≜ & {\left[\begin{array}{cccc} {ℷ}_{1}\left(k\right) & {ℷ}_{2}\left(k\right) & \cdots \; & {ℷ}_{n_{u}}\left(k\right) \end{array}\right]}^{T}\left(ℷ=\bar{u},\bar{\bar{u}},{\tilde{q}}_{\bar{u}},\overset{˘}{\bar{u}},\tilde{\bar{u}},{\tilde{r}}_{\bar{u}}\right). \end{array}$$

Based on (10) and (19), the statistical properties of ${\tilde{q}}_{\bar{u}}\left(k\right)$ and ${\tilde{r}}_{\bar{u}}\left(k\right)$ are denoted below:

(27) $$\begin{array}{cc} \; & E\left\{{\tilde{q}}_{\bar{u}}\left(k\right)\right\}=0,V\left\{{\tilde{q}}_{\bar{u}}\left(k\right)\right\}\leq \theta^{2}/4I_{\left(n_{u}\right)},\\ \; & E\left\{{\tilde{r}}_{\bar{u}}\left(k\right)\right\}=0,V\left\{{\tilde{r}}_{\bar{u}}\left(k\right)\right\}=\left(1/{\left(1-2\bar{϶}\right)}^{2}\right){ℑ}^{2}\pi I_{\left(n_{u}\right)}. \end{array}$$

The DoS attacks may also appear in the controller-to-actuator channel, then the actually received signal by the actuator is expressed by:

(28) $$\begin{array}{c} u\left(k\right)=\left(1-\beta_{2}\left(k\right)\right)\tilde{\bar{u}}\left(k\right) \end{array}$$

where $\beta_{2}\left(k\right)$ is a Bernoulli-distributed random variable with $\mathrm{Prob}\left\{\beta_{2}\left(k\right)=1\right\}=\bar{\beta }$ and $\mathrm{Prob}\left\{\beta_{2}\left(k\right)=0\right\}=1-\bar{\beta }$ with $\bar{\beta }$ being defined in (24).

### 2.4. The Closed-Loop System

Setting $e\left(k\right)≜\eta \left(k\right)-\hat{x}\left(k\right)$ as the estimation error, the following expression is acquired from (4) and (25):

(29) $$\begin{array}{cc} e(k+1)= & (\bar{A}-L\bar{C})e\left(k\right)+\left(\bar{\alpha }\Delta {\bar{A}}_{1}+\bar{\beta }L\bar{C}\right)\eta \left(k\right)+\tilde{\alpha }\left(k\right)\Delta {\bar{A}}_{1}\eta \left(k\right)+{\tilde{\beta }}_{1}\left(k\right)L\bar{C}\eta \left(k\right)\\ \; & +\bar{G}w\left(k\right)+I_{f}f\left(k,x\left(k\right)\right)-\left(1-\bar{\beta }\right)LDv\left(k\right)+{\tilde{\beta }}_{1}\left(k\right)LDv\left(k\right)-\left(1-\bar{\beta }\right)L{\tilde{q}}_{y}\left(k\right)\\ \; & +{\tilde{\beta }}_{1}\left(k\right)L{\tilde{q}}_{y}\left(k\right)-\left(1-\bar{\beta }\right)L{\tilde{r}}_{y}\left(k\right)+{\tilde{\beta }}_{1}\left(k\right)L{\tilde{r}}_{y}\left(k\right) \end{array}$$

where $\tilde{\alpha }\left(k\right)≜\alpha \left(k\right)-\bar{\alpha }$ and ${\tilde{\beta }}_{1}\left(k\right)≜\beta_{1}\left(k\right)-\bar{\beta }$.

In terms of (4), (25), (26) and (28), the closed-loop system is expressed by the following form:

(30) $$\begin{array}{cc} \eta (k+1)= & \bar{A}\eta \left(k\right)+\bar{\alpha }\Delta \bar{A_{1}}\eta \left(k\right)+\left(1-\bar{\beta }\right)\bar{B}\left(K_{P}+K_{D}\right)\eta \left(k\right)+\tilde{\alpha }\left(k\right)\Delta \bar{A_{1}}\eta \left(k\right)\\ \; & -{\tilde{\beta }}_{2}\left(k\right)\bar{B}\left(K_{P}+K_{D}\right)\eta \left(k\right)-\left(1-\bar{\beta }\right)\bar{B}\left(K_{P}+K_{D}\right)e\left(k\right)+{\tilde{\beta }}_{2}\left(k\right)\bar{B}\left(K_{P}+K_{D}\right)e\left(k\right)\\ \; & +\left(1-\bar{\beta }\right)\bar{B}K_{I}\sum_{l=k-N}^{k-1}\eta \left(l\right)-\left(1-\bar{\beta }\right)\bar{B}K_{I}\sum_{l=k-N}^{k-1}e\left(l\right)-{\tilde{\beta }}_{2}\left(k\right)\bar{B}K_{I}\sum_{l=k-N}^{k-1}\eta \left(l\right)\\ \; & +{\tilde{\beta }}_{2}\left(k\right)\bar{B}K_{I}\sum_{l=k-N}^{k-1}e\left(l\right)-\left(1-\bar{\beta }\right)\bar{B}K_{D}\eta \left(k-1\right)+\left(1-\bar{\beta }\right)\bar{B}K_{D}e\left(k-1\right)\\ \; & +{\tilde{\beta }}_{2}\left(k\right)\bar{B}K_{D}\eta \left(k-1\right)-{\tilde{\beta }}_{2}\left(k\right)\bar{B}K_{D}e\left(k-1\right)+I_{f}f\left(k,I_{f}^{T}\eta \left(k\right)\right)\\ \; & +\bar{G}w\left(k\right)+\left(1-\bar{\beta }\right)\bar{B}{\tilde{q}}_{\bar{u}}\left(k\right)-{\tilde{\beta }}_{2}\left(k\right)\bar{B}{\tilde{q}}_{\bar{u}}\left(k\right)+\left(1-\bar{\beta }\right)\bar{B}{\tilde{r}}_{\bar{u}}\left(k\right)-{\tilde{\beta }}_{2}\left(k\right)\bar{B}{\tilde{r}}_{\bar{u}}\left(k\right) \end{array}$$

where ${\tilde{\beta }}_{2}\left(k\right)≜\beta_{2}\left(k\right)-\bar{\beta }$.

Letting $\bar{\eta }\left(k\right)≜{\left[\begin{array}{cc} \eta^{T}\left(k\right) & e^{T}\left(k\right) \end{array}\right]}^{T}$, we derive the expression of the closed-loop augmented system based on (29) and (30) as follows:

(31) $$\begin{array}{cc} \bar{\eta }\left(k+1\right)= & \left(\overset{˘}{A}+\bar{\alpha }\Delta {\overset{˘}{A}}_{1}\right)\bar{\eta }\left(k\right)+\tilde{\alpha }\left(k\right)\Delta {\overset{˘}{A}}_{1}\bar{\eta }\left(k\right)+{\tilde{\beta }}_{1}\left(k\right){\bar{L}}_{C}\bar{\eta }\left(k\right)+{\tilde{\beta }}_{2}\left(k\right){\overset{˘}{B}}_{L}\bar{\eta }\left(k\right)\\ \; & +{\bar{I}}_{f1}f\left(k,I_{f}^{T}\bar{I}\bar{\eta }\left(k\right)\right)+{\bar{G}}_{1}w\left(k\right)+\left(1-\bar{\beta }\right){\overset{˘}{B}}_{D}{\bar{\eta }}^{*}\left(k\right)-{\tilde{\beta }}_{2}\left(k\right){\overset{˘}{B}}_{D}{\bar{\eta }}^{*}\left(k\right)\\ \; & -\left(1-\bar{\beta }\right){\bar{L}}_{D}v\left(k\right)+{\tilde{\beta }}_{1}\left(k\right){\bar{L}}_{D}v\left(k\right)-\left(1-\bar{\beta }\right){\bar{L}}_{1}{\tilde{q}}_{y}\left(k\right)+{\tilde{\beta }}_{1}\left(k\right){\bar{L}}_{1}{\tilde{q}}_{y}\left(k\right)\\ \; & -\left(1-\bar{\beta }\right){\bar{L}}_{1}{\tilde{r}}_{y}\left(k\right)+{\tilde{\beta }}_{1}\left(k\right){\bar{L}}_{1}{\tilde{r}}_{y}\left(k\right)+\left(1-\bar{\beta }\right){\bar{B}}_{1}{\tilde{q}}_{\bar{u}}\left(k\right)\\ \; & -{\tilde{\beta }}_{2}\left(k\right){\bar{B}}_{1}{\tilde{q}}_{\bar{u}}\left(k\right)-{\tilde{\beta }}_{2}\left(k\right){\bar{B}}_{1}{\tilde{r}}_{\bar{u}}\left(k\right)+\left(1-\bar{\beta }\right){\bar{B}}_{1}{\tilde{r}}_{\bar{u}}\left(k\right)\\ z(k)= & \tilde{H}\bar{\eta }\left(k\right) \end{array}$$

where

$$\begin{array}{cc} \; & \overset{˘}{A}≜\left[\begin{array}{cc} \bar{A}+\left(1-\bar{\beta }\right)\bar{B}\left(K_{P}+K_{D}\right) & -\left(1-\bar{\beta }\right)\bar{B}\left(K_{P}+K_{D}\right)\\ \bar{\beta }L\bar{C} & \bar{A}-L\bar{C} \end{array}\right],\Delta {\overset{˘}{A}}_{1}≜\left[\begin{array}{cc} \Delta {\bar{A}}_{1} & 0\\ \Delta {\bar{A}}_{1} & 0 \end{array}\right],\\ \; & {\bar{B}}_{1}≜\left[\begin{array}{c} \bar{B}\\ 0 \end{array}\right],{\overset{˘}{B}}_{L}≜\left[\begin{array}{cc} -\bar{B}\left(K_{P}+K_{D}\right) & \bar{B}\left(K_{P}+K_{D}\right)\\ 0 & 0 \end{array}\right],{\bar{L}}_{C}≜\left[\begin{array}{cc} 0 & 0\\ L\bar{C} & 0 \end{array}\right],{\overset{˘}{B}}_{D}≜\left[\begin{array}{c} \bar{B}{\bar{B}}_{K}\\ 0 \end{array}\right],\\ \; & {\bar{B}}_{K}≜\left[{\bar{K}}_{I}-{\bar{K}}_{D}\;\;\underset{N-1}{\underset{︸}{\begin{array}{ccc} {\bar{K}}_{I} & \cdots & {\bar{K}}_{I} \end{array}}}\right],{\bar{G}}_{1}≜\left[\begin{array}{c} \bar{G}\\ \bar{G} \end{array}\right],{\bar{L}}_{1}≜\left[\begin{array}{c} 0\\ L \end{array}\right],{\bar{L}}_{D}≜\left[\begin{array}{c} 0\\ LD \end{array}\right],{\bar{I}}_{f1}≜\left[\begin{array}{c} I_{f}\\ I_{f} \end{array}\right],\\ \; & {\bar{K}}_{D}≜\left[\begin{array}{cc} K_{D} & -K_{D} \end{array}\right],{\bar{K}}_{I}≜\left[\begin{array}{cc} K_{I} & -K_{I} \end{array}\right],\tilde{H}≜\left[\begin{array}{cc} \bar{H} & 0 \end{array}\right],\bar{I}≜\left[\begin{array}{cc} I & 0 \end{array}\right],\\ \; & {\bar{\eta }}^{*}\left(k\right)≜{\left[\begin{array}{cccc} {\bar{\eta }}^{T}\left(k-1\right) & {\bar{\eta }}^{T}\left(k-2\right) & \cdots & {\bar{\eta }}^{T}\left(k-N\right) \end{array}\right]}^{T}. \end{array}$$

**Definition** **1.** *For given scalars c>0, l≥0 and 0≤h<1, system (31) has EUBMS performance if the following inequality holds:*

(32) $$\begin{array}{c} E\{∥\bar{\eta }{\left(k\right)∥}^{2}\}\leq ch^{k}\max_{i\leq 0}E\{∥\bar{\eta }\left(i\right){∥}^{2}\}+l,\forall k\geq 0 \end{array}$$

*for any solution $\bar{\eta }\left(k\right)$ with the initial condition $\bar{\eta }\left(i\right)$ (i≤0), where l is an ultimate upper bound in mean square of (31).*

The object of this paper is to design an observer-based PID controller for uncertain nonlinear systems (1) with integral measurements and DoS attacks using a BES such that the following two performance requirements are met:

(1) under the influence from stochastic noises w(k) and v(k), quantization errors ${\tilde{q}}_{y}\left(k\right)$ and ${\tilde{q}}_{\bar{u}}\left(k\right)$, and random bit errors ${\tilde{r}}_{y}\left(k\right)$ and ${\tilde{r}}_{\bar{u}}\left(k\right)$, the closed-loop system (31) realizes EUBMS performance;
(2) the controlled output z(k) has an ultimate upper bound in mean square, which is bounded and such a bound is minimized by designing appropriate gain parameters $K_{P}$, $K_{I}$, $K_{D}$ and *L* of controller and observer.

**Figure 1:**
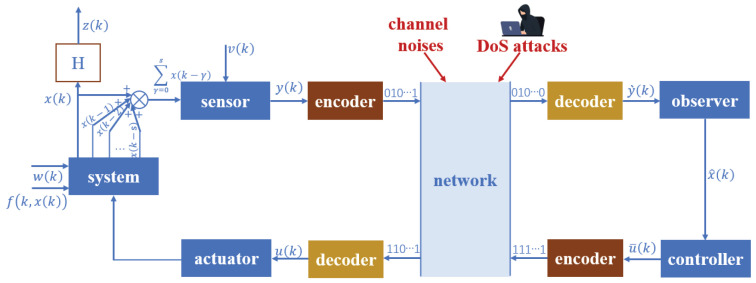
The diagram of control issue in this paper.

Figure 1 indicates the research structure of this paper.

## 3. Main Results

**Lemma** **2** **([**57**]).** *For a scalar ℘>0, real vectors $M\in R^{n}$ and $N\in R^{n}$, and a matrix $Q>0\in R^{n\times n}$, the following matrix inequality:*

(33) $$\begin{array}{c} M^{T}QN+N^{T}QM\leq ℘M^{T}QM+{℘}^{-1}N^{T}QN \end{array}$$

*holds.*

**Theorem** **1.** *Given scalars $h_{0}>1$ and ${℘}_{𝚥}>0\;$(𝚥=1,2,…,8) and gain matrices L, $K_{P}$, $K_{I}$ and $K_{D}$, the closed-loop system (31) satisfies EUBMS performance, if there exist matrices P>0 and $Q_{j}>0\;$(j=1,2,…,N) ensuring the following inequality holds:*

(34) $$\begin{array}{c} \Pi ≜\left[\begin{array}{cc} \Pi_{11} & \Pi_{12}\\ {}* & \Pi_{22} \end{array}\right]<0 \end{array}$$

*where*

$$\begin{array}{cc} \Pi_{11}≜ & -P+{\left(\overset{˘}{A}+\bar{\alpha }\Delta {\overset{˘}{A}}_{1}\right)}^{T}P\left(\overset{˘}{A}+\bar{\alpha }\Delta {\overset{˘}{A}}_{1}\right)+{\tilde{\alpha }}^{2}\Delta {\overset{˘}{A}}_{1}^{T}P\Delta {\overset{˘}{A}}_{1}+{\tilde{\beta }}^{2}{\bar{L}}_{C}^{T}P{\bar{L}}_{C}+{\tilde{\beta }}^{2}{\overset{˘}{B}}_{L}^{T}P{\overset{˘}{B}}_{L}\\ \; & +\sum_{l=1}^{\xi }\mathrm{tr}\left(\iota_{l}^{T}{\bar{I}}_{f1}^{T}P{\bar{I}}_{f1}\iota_{l}\right){\bar{I}}^{T}I_{f}\Gamma_{l}I_{f}^{T}\bar{I}+\sum_{j=1}^{N}Q_{j},\\ \Pi_{22}≜ & -Q+{\left(1-\bar{\beta }\right)}^{2}{\overset{˘}{B}}_{D}^{T}P{\overset{˘}{B}}_{D}+{\tilde{\beta }}^{2}{\overset{˘}{B}}_{D}^{T}P{\overset{˘}{B}}_{D},Q≜\mathrm{diag}\left\{Q_{1},Q_{2},\cdots ,Q_{N}\right\},\\ \Pi_{12}≜ & \left(1-\bar{\beta }\right){\left(\overset{˘}{A}+\bar{\alpha }\Delta {\overset{˘}{A}}_{1}\right)}^{T}P{\overset{˘}{B}}_{D}-{\tilde{\beta }}^{2}{\overset{˘}{B}}_{L}^{T}P{\overset{˘}{B}}_{D},{\tilde{\alpha }}^{2}≜\bar{\alpha }\left(1-\bar{\alpha }\right),{\tilde{\beta }}^{2}≜\bar{\beta }\left(1-\bar{\beta }\right). \end{array}$$

**Proof of Theorem** **1.** Define Lyapunov-Krasovskii function as follows:

(35) $$\begin{array}{c} V\left(k\right)≜V_{1}\left(k\right)+V_{2}\left(k\right) \end{array}$$

where

$$\begin{array}{c} V_{1}\left(k\right)≜{\bar{\eta }}^{T}\left(k\right)P\bar{\eta }\left(k\right),V_{2}\left(k\right)≜\sum_{j=1}^{N}\sum_{i=k-j}^{k-1}{\bar{\eta }}^{T}\left(i\right)Q_{j}\bar{\eta }\left(i\right). \end{array}$$

Computing the expectation of the difference of V(k) along the trajectory of (31) and considering the expectation properties (22) and (27), we get

(36) $$\begin{array}{cc} \; & E\{\Delta V_{1}\left(k\right)\}\\ = & E\{{\bar{\eta }}^{T}\left(k+1\right)P\bar{\eta }\left(k+1\right)-{\bar{\eta }}^{T}\left(k\right)P\bar{\eta }\left(k\right)\}\\ = & E\{{\bar{\eta }}^{T}\left(k\right){\left(\overset{˘}{A}+\bar{\alpha }\Delta {\overset{˘}{A}}_{1}\right)}^{T}P\left(\overset{˘}{A}+\bar{\alpha }\Delta {\overset{˘}{A}}_{1}\right)\bar{\eta }\left(k\right)+2{\bar{\eta }}^{T}\left(k\right){\left(\overset{˘}{A}+\bar{\alpha }\Delta {\overset{˘}{A}}_{1}\right)}^{T}P\left(1-\bar{\beta }\right){\overset{˘}{B}}_{D}{\bar{\eta }}^{*}\left(k\right)\\ \; & +\left(1-\bar{\beta }\right){\bar{\eta }}^{*T}\left(k\right){\overset{˘}{B}}_{D}^{T}P\left(1-\bar{\beta }\right){\overset{˘}{B}}_{D}{\bar{\eta }}^{*}\left(k\right)+\tilde{\alpha }\left(k\right){\bar{\eta }}^{T}\left(k\right)\Delta {\overset{˘}{A}}_{1}^{T}P\tilde{\alpha }\left(k\right)\Delta {\overset{˘}{A}}_{1}\bar{\eta }\left(k\right)\\ \; & +{\tilde{\beta }}_{1}\left(k\right){\bar{\eta }}^{T}\left(k\right){\bar{L}}_{C}^{T}P{\tilde{\beta }}_{1}\left(k\right){\bar{L}}_{C}\bar{\eta }\left(k\right)+{\tilde{\beta }}_{2}\left(k\right){\bar{\eta }}^{T}\left(k\right){\overset{˘}{B}}_{L}^{T}P{\tilde{\beta }}_{2}\left(k\right){\overset{˘}{B}}_{L}\bar{\eta }\left(k\right)\\ \; & -2{\tilde{\beta }}_{2}\left(k\right){\bar{\eta }}^{T}\left(k\right){\overset{˘}{B}}_{L}^{T}P{\tilde{\beta }}_{2}\left(k\right){\overset{˘}{B}}_{D}{\bar{\eta }}^{*}\left(k\right)+w^{T}\left(k\right){\bar{G}}_{1}^{T}P{\bar{G}}_{1}w\left(k\right)\\ \; & +{\tilde{\beta }}_{2}\left(k\right){\bar{\eta }}^{*T}\left(k\right){\overset{˘}{B}}_{D}^{T}P{\tilde{\beta }}_{2}\left(k\right){\overset{˘}{B}}_{D}{\bar{\eta }}^{*}\left(k\right)+f^{T}\left(k,I_{f}^{T}\bar{I}\bar{\eta }\left(k\right)\right){\bar{I}}_{f1}^{T}P{\bar{I}}_{f1}f\left(k,I_{f}^{T}\bar{I}\bar{\eta }\left(k\right)\right)\\ \; & +\left(1-\bar{\beta }\right)v^{T}\left(k\right){\bar{L}}_{D}^{T}P\left(1-\bar{\beta }\right){\bar{L}}_{D}v\left(k\right)+2\left(1-\bar{\beta }\right)v^{T}\left(k\right){\bar{L}}_{D}^{T}P\left(1-\bar{\beta }\right){\bar{L}}_{1}{\tilde{q}}_{y}\left(k\right)\\ \; & +2\left(1-\bar{\beta }\right)v^{T}\left(k\right){\bar{L}}_{D}^{T}P\left(1-\bar{\beta }\right){\bar{L}}_{1}{\tilde{r}}_{y}\left(k\right)+\left(1-\bar{\beta }\right){\tilde{q}}_{y}^{T}\left(k\right){\bar{L}}_{1}^{T}P\left(1-\bar{\beta }\right){\bar{L}}_{1}{\tilde{q}}_{y}\left(k\right)\\ \; & +2\left(1-\bar{\beta }\right){\tilde{q}}_{y}^{T}\left(k\right){\bar{L}}_{1}^{T}P\left(1-\bar{\beta }\right){\bar{L}}_{1}{\tilde{r}}_{y}\left(k\right)+\left(1-\bar{\beta }\right){\tilde{r}}_{y}^{T}\left(k\right){\bar{L}}_{1}^{T}P\left(1-\bar{\beta }\right){\bar{L}}_{1}{\tilde{r}}_{y}\left(k\right)\\ \; & +{\tilde{\beta }}_{1}\left(k\right)v^{T}\left(k\right){\bar{L}}_{D}^{T}P{\tilde{\beta }}_{1}\left(k\right){\bar{L}}_{D}v\left(k\right)+2{\tilde{\beta }}_{1}\left(k\right)v^{T}\left(k\right){\bar{L}}_{D}^{T}P{\tilde{\beta }}_{1}\left(k\right){\bar{L}}_{1}{\tilde{q}}_{y}\left(k\right)\\ \; & +2{\tilde{\beta }}_{1}\left(k\right)v^{T}\left(k\right){\bar{L}}_{D}^{T}P{\tilde{\beta }}_{1}\left(k\right){\bar{L}}_{1}{\tilde{r}}_{y}\left(k\right)+{\tilde{\beta }}_{1}\left(k\right){\tilde{q}}_{y}^{T}\left(k\right){\bar{L}}_{1}^{T}P{\tilde{\beta }}_{1}\left(k\right){\bar{L}}_{1}{\tilde{q}}_{y}\left(k\right)\\ \; & +2{\tilde{\beta }}_{1}\left(k\right){\tilde{q}}_{y}^{T}\left(k\right){\bar{L}}_{1}^{T}P{\tilde{\beta }}_{1}\left(k\right){\bar{L}}_{1}{\tilde{r}}_{y}\left(k\right)+{\tilde{\beta }}_{1}\left(k\right){\tilde{r}}_{y}^{T}\left(k\right){\bar{L}}_{1}^{T}P{\tilde{\beta }}_{1}\left(k\right){\bar{L}}_{1}{\tilde{r}}_{y}\left(k\right)\\ \; & +\left(1-\bar{\beta }\right){\tilde{q}}_{\bar{u}}^{T}\left(k\right){\bar{B}}_{1}^{T}P\left(1-\bar{\beta }\right){\bar{B}}_{1}{\tilde{q}}_{\bar{u}}\left(k\right)+2\left(1-\bar{\beta }\right){\tilde{q}}_{\bar{u}}^{T}\left(k\right){\bar{B}}_{1}^{T}P\left(1-\bar{\beta }\right){\bar{B}}_{1}{\tilde{r}}_{\bar{u}}\left(k\right)\\ \; & +\left(1-\bar{\beta }\right){\tilde{r}}_{\bar{u}}^{T}\left(k\right){\bar{B}}_{1}^{T}P\left(1-\bar{\beta }\right){\bar{B}}_{1}{\tilde{r}}_{\bar{u}}\left(k\right)+{\tilde{\beta }}_{2}\left(k\right){\tilde{q}}_{\bar{u}}^{T}\left(k\right){\bar{B}}_{1}^{T}P{\tilde{\beta }}_{2}\left(k\right){\bar{B}}_{1}{\tilde{q}}_{\bar{u}}\left(k\right)\\ \; & +2{\tilde{\beta }}_{2}\left(k\right){\tilde{q}}_{\bar{u}}^{T}\left(k\right){\bar{B}}_{1}^{T}P{\tilde{\beta }}_{2}\left(k\right){\bar{B}}_{1}{\tilde{r}}_{\bar{u}}\left(k\right)+{\tilde{\beta }}_{2}\left(k\right){\tilde{r}}_{\bar{u}}^{T}\left(k\right){\bar{B}}_{1}^{T}P{\tilde{\beta }}_{2}\left(k\right){\bar{B}}_{1}{\tilde{r}}_{\bar{u}}\left(k\right)-{\bar{\eta }}^{T}\left(k\right)P\bar{\eta }\left(k\right)\}\\ = & E\{{\bar{\eta }}^{T}\left(k\right){\left(\overset{˘}{A}+\bar{\alpha }\Delta {\overset{˘}{A}}_{1}\right)}^{T}P\left(\overset{˘}{A}+\bar{\alpha }\Delta {\overset{˘}{A}}_{1}\right)\bar{\eta }\left(k\right)+2\left(1-\bar{\beta }\right){\bar{\eta }}^{T}\left(k\right){\left(\overset{˘}{A}+\bar{\alpha }\Delta {\overset{˘}{A}}_{1}\right)}^{T}P{\overset{˘}{B}}_{D}{\bar{\eta }}^{*}\left(k\right)\\ \; & +{\left(1-\bar{\beta }\right)}^{2}{\bar{\eta }}^{*T}\left(k\right){\overset{˘}{B}}_{D}^{T}P{\overset{˘}{B}}_{D}{\bar{\eta }}^{*}\left(k\right)+{\tilde{\alpha }}^{2}{\bar{\eta }}^{T}\left(k\right)\Delta {\overset{˘}{A}}_{1}^{T}P\Delta {\overset{˘}{A}}_{1}\bar{\eta }\left(k\right)+{\tilde{\beta }}^{2}{\bar{\eta }}^{T}\left(k\right){\bar{L}}_{C}^{T}P{\bar{L}}_{C}\bar{\eta }\left(k\right)\\ \; & +{\tilde{\beta }}^{2}{\bar{\eta }}^{T}\left(k\right){\overset{˘}{B}}_{L}^{T}P{\overset{˘}{B}}_{L}\bar{\eta }\left(k\right)-2{\tilde{\beta }}^{2}{\bar{\eta }}^{T}\left(k\right){\overset{˘}{B}}_{L}^{T}P{\overset{˘}{B}}_{D}{\bar{\eta }}^{*}\left(k\right)+w^{T}\left(k\right){\bar{G}}_{1}^{T}P{\bar{G}}_{1}w\left(k\right)\\ \; & +{\tilde{\beta }}^{2}{\bar{\eta }}^{*T}\left(k\right){\overset{˘}{B}}_{D}^{T}P{\overset{˘}{B}}_{D}{\bar{\eta }}^{*}\left(k\right)+f^{T}\left(k,I_{f}^{T}\bar{I}\bar{\eta }\left(k\right)\right){\bar{I}}_{f1}^{T}P{\bar{I}}_{f1}f\left(k,I_{f}^{T}\bar{I}\bar{\eta }\left(k\right)\right)\\ \; & +{\left(1-\bar{\beta }\right)}^{2}v^{T}\left(k\right){\bar{L}}_{D}^{T}P{\bar{L}}_{D}v\left(k\right)+2{\left(1-\bar{\beta }\right)}^{2}v^{T}\left(k\right){\bar{L}}_{D}^{T}P{\bar{L}}_{1}{\tilde{q}}_{y}\left(k\right)\\ \; & +2{\left(1-\bar{\beta }\right)}^{2}v^{T}\left(k\right){\bar{L}}_{D}^{T}P{\bar{L}}_{1}{\tilde{r}}_{y}\left(k\right)+{\left(1-\bar{\beta }\right)}^{2}{\tilde{q}}_{y}^{T}\left(k\right){\bar{L}}_{1}^{T}P{\bar{L}}_{1}{\tilde{q}}_{y}\left(k\right)\\ \; & +2{\left(1-\bar{\beta }\right)}^{2}{\tilde{q}}_{y}^{T}\left(k\right){\bar{L}}_{1}^{T}P{\bar{L}}_{1}{\tilde{r}}_{y}\left(k\right)+{\left(1-\bar{\beta }\right)}^{2}{\tilde{r}}_{y}^{T}\left(k\right){\bar{L}}_{1}^{T}P{\bar{L}}_{1}{\tilde{r}}_{y}\left(k\right)\\ \; & +{\tilde{\beta }}^{2}v^{T}\left(k\right){\bar{L}}_{D}^{T}P{\bar{L}}_{D}v\left(k\right)+2{\tilde{\beta }}^{2}v^{T}\left(k\right){\bar{L}}_{D}^{T}P{\bar{L}}_{1}{\tilde{q}}_{y}\left(k\right)\\ \; & +2{\tilde{\beta }}^{2}v^{T}\left(k\right){\bar{L}}_{D}^{T}P{\bar{L}}_{1}{\tilde{r}}_{y}\left(k\right)+{\tilde{\beta }}^{2}{\tilde{q}}_{y}^{T}\left(k\right){\bar{L}}_{1}^{T}P{\bar{L}}_{1}{\tilde{q}}_{y}\left(k\right)\\ \; & +2{\tilde{\beta }}^{2}{\tilde{q}}_{y}^{T}\left(k\right){\bar{L}}_{1}^{T}P{\bar{L}}_{1}{\tilde{r}}_{y}\left(k\right)+{\tilde{\beta }}^{2}{\tilde{r}}_{y}^{T}\left(k\right){\bar{L}}_{1}^{T}P{\bar{L}}_{1}{\tilde{r}}_{y}\left(k\right)\\ \; & +{\left(1-\bar{\beta }\right)}^{2}{\tilde{q}}_{\bar{u}}^{T}\left(k\right){\bar{B}}_{1}^{T}P{\bar{B}}_{1}{\tilde{q}}_{\bar{u}}\left(k\right)+2{\left(1-\bar{\beta }\right)}^{2}{\tilde{q}}_{\bar{u}}^{T}\left(k\right){\bar{B}}_{1}^{T}P{\bar{B}}_{1}{\tilde{r}}_{\bar{u}}\left(k\right)\\ \; & +{\left(1-\bar{\beta }\right)}^{2}{\tilde{r}}_{\bar{u}}^{T}\left(k\right){\bar{B}}_{1}^{T}P{\bar{B}}_{1}{\tilde{r}}_{\bar{u}}\left(k\right)+{\tilde{\beta }}^{2}{\tilde{q}}_{\bar{u}}^{T}\left(k\right){\bar{B}}_{1}^{T}P{\bar{B}}_{1}{\tilde{q}}_{\bar{u}}\left(k\right)\\ \; & +2{\tilde{\beta }}^{2}{\tilde{q}}_{\bar{u}}^{T}\left(k\right){\bar{B}}_{1}^{T}P{\bar{B}}_{1}{\tilde{r}}_{\bar{u}}\left(k\right)+{\tilde{\beta }}^{2}{\tilde{r}}_{\bar{u}}^{T}\left(k\right){\bar{B}}_{1}^{T}P{\bar{B}}_{1}{\tilde{r}}_{\bar{u}}\left(k\right)-{\bar{\eta }}^{T}\left(k\right)P\bar{\eta }\left(k\right)\} \end{array}$$

and

(37) $$\begin{array}{cc} E\{\Delta V_{2}\left(k\right)\}\; & =E\left\{\sum_{j=1}^{N}(\sum_{i=k+1-j}^{k}{\bar{\eta }}^{T}\left(i\right)Q_{j}\bar{\eta }\left(i\right)-\sum_{i=k-j}^{k-1}{\bar{\eta }}^{T}\left(i\right)Q_{j}\bar{\eta }\left(i\right))\right\}\\ \; & =E\left\{\sum_{j=1}^{N}\left({\bar{\eta }}^{T}\left(k\right)Q_{j}\bar{\eta }\left(k\right)-{\bar{\eta }}^{T}\left(k-j\right)Q_{j}\bar{\eta }\left(k-j\right)\right)\right\}\\ \; & =E\left\{\sum_{j=1}^{N}{\bar{\eta }}^{T}\left(k\right)Q_{j}\bar{\eta }\left(k\right)-\sum_{j=1}^{N}{\bar{\eta }}^{T}\left(k-j\right)Q_{j}\bar{\eta }\left(k-j\right)\right\}\\ \; & =E\left\{{\bar{\eta }}^{T}\left(k\right)\sum_{j=1}^{N}Q_{j}\bar{\eta }\left(k\right)-{\bar{\eta }}^{*T}\left(k\right)Q{\bar{\eta }}^{*}\left(k\right)\right\}. \end{array}$$

By employing Lemma 2, we have the following inequalities:

(38) $$\begin{array}{cc} \; & 2{\left(1-\bar{\beta }\right)}^{2}v^{T}\left(k\right){\bar{L}}_{D}^{T}P{\bar{L}}_{1}{\tilde{q}}_{y}\left(k\right)\leq {℘}_{1}{\left(1-\bar{\beta }\right)}^{2}v^{T}\left(k\right){\bar{L}}_{D}^{T}P{\bar{L}}_{D}v\left(k\right)+{℘}_{1}^{-1}{\left(1-\bar{\beta }\right)}^{2}{\tilde{q}}_{y}^{T}\left(k\right){\bar{L}}_{1}^{T}P{\bar{L}}_{1}{\tilde{q}}_{y}\left(k\right),\\ \; & 2{\left(1-\bar{\beta }\right)}^{2}v^{T}\left(k\right){\bar{L}}_{D}^{T}P{\bar{L}}_{1}{\tilde{r}}_{y}\left(k\right)\leq {℘}_{2}{\left(1-\bar{\beta }\right)}^{2}v^{T}\left(k\right){\bar{L}}_{D}^{T}P{\bar{L}}_{D}v\left(k\right)+{℘}_{2}^{-1}{\left(1-\bar{\beta }\right)}^{2}{\tilde{r}}_{y}^{T}\left(k\right){\bar{L}}_{1}^{T}P{\bar{L}}_{1}{\tilde{r}}_{y}\left(k\right),\\ \; & 2{\left(1-\bar{\beta }\right)}^{2}{\tilde{q}}_{y}^{T}\left(k\right){\bar{L}}_{1}^{T}P{\bar{L}}_{1}{\tilde{r}}_{y}\left(k\right)\leq {℘}_{3}{\left(1-\bar{\beta }\right)}^{2}{\tilde{q}}_{y}^{T}\left(k\right){\bar{L}}_{1}^{T}P{\bar{L}}_{1}{\tilde{q}}_{y}\left(k\right)+{℘}_{3}^{-1}{\left(1-\bar{\beta }\right)}^{2}{\tilde{r}}_{y}^{T}\left(k\right){\bar{L}}_{1}^{T}P{\bar{L}}_{1}{\tilde{r}}_{y}\left(k\right),\\ \; & 2{\tilde{\beta }}^{2}v^{T}\left(k\right){\bar{L}}_{D}^{T}P{\bar{L}}_{1}{\tilde{q}}_{y}\left(k\right)\leq {℘}_{4}{\tilde{\beta }}^{2}v^{T}\left(k\right){\bar{L}}_{D}^{T}P{\bar{L}}_{D}v\left(k\right)+{℘}_{4}^{-1}{\tilde{\beta }}^{2}{\tilde{q}}_{y}^{T}\left(k\right){\bar{L}}_{1}^{T}P{\bar{L}}_{1}{\tilde{q}}_{y}\left(k\right),\\ \; & 2{\tilde{\beta }}^{2}v^{T}\left(k\right){\bar{L}}_{D}^{T}P{\bar{L}}_{1}{\tilde{r}}_{y}\left(k\right)\leq {℘}_{5}{\tilde{\beta }}^{2}v^{T}\left(k\right){\bar{L}}_{D}^{T}P{\bar{L}}_{D}v\left(k\right)+{℘}_{5}^{-1}{\tilde{\beta }}^{2}{\tilde{r}}_{y}^{T}\left(k\right){\bar{L}}_{1}^{T}P{\bar{L}}_{1}{\tilde{r}}_{y}\left(k\right),\\ \; & 2{\tilde{\beta }}^{2}{\tilde{q}}_{y}^{T}\left(k\right){\bar{L}}_{1}^{T}P{\bar{L}}_{1}{\tilde{r}}_{y}\left(k\right)\leq {℘}_{6}{\tilde{\beta }}^{2}{\tilde{q}}_{y}^{T}\left(k\right){\bar{L}}_{1}^{T}P{\bar{L}}_{1}{\tilde{q}}_{y}\left(k\right)+{℘}_{6}^{-1}{\tilde{\beta }}^{2}{\tilde{r}}_{y}^{T}\left(k\right){\bar{L}}_{1}^{T}P{\bar{L}}_{1}{\tilde{r}}_{y}\left(k\right),\\ \; & 2{\left(1-\bar{\beta }\right)}^{2}{\tilde{q}}_{\bar{u}}^{T}\left(k\right){\bar{B}}_{1}^{T}P{\bar{B}}_{1}{\tilde{r}}_{\bar{u}}\left(k\right)\leq {℘}_{7}{\left(1-\bar{\beta }\right)}^{2}{\tilde{q}}_{\bar{u}}^{T}\left(k\right){\bar{B}}_{1}^{T}P{\bar{B}}_{1}{\tilde{q}}_{\bar{u}}\left(k\right)+{℘}_{7}^{-1}{\left(1-\bar{\beta }\right)}^{2}{\tilde{r}}_{\bar{u}}^{T}\left(k\right){\bar{B}}_{1}^{T}P{\bar{B}}_{1}{\tilde{r}}_{\bar{u}}\left(k\right),\\ \; & 2{\tilde{\beta }}^{2}{\tilde{q}}_{\bar{u}}^{T}\left(k\right){\bar{B}}_{1}^{T}P{\bar{B}}_{1}{\tilde{r}}_{\bar{u}}\left(k\right)\leq {℘}_{8}{\tilde{\beta }}^{2}{\tilde{r}}_{\bar{u}}^{T}\left(k\right){\bar{B}}_{1}^{T}P{\bar{B}}_{1}{\tilde{r}}_{\bar{u}}\left(k\right)+{℘}_{8}^{-1}{\tilde{\beta }}^{2}{\tilde{q}}_{\bar{u}}^{T}\left(k\right){\bar{B}}_{1}^{T}P{\bar{B}}_{1}{\tilde{q}}_{\bar{u}}\left(k\right). \end{array}$$

Combining (38) and (36), we yield that

(39) $$\begin{array}{cc} \; & E\{\Delta V_{1}\left(k\right)\}\\ \leq & E\{{\bar{\eta }}^{T}\left(k\right){\left(\overset{˘}{A}+\bar{\alpha }\Delta {\overset{˘}{A}}_{1}\right)}^{T}P\left(\overset{˘}{A}+\bar{\alpha }\Delta {\overset{˘}{A}}_{1}\right)\bar{\eta }\left(k\right)+2\left(1-\bar{\beta }\right){\bar{\eta }}^{T}\left(k\right){\left(\overset{˘}{A}+\bar{\alpha }\Delta {\overset{˘}{A}}_{1}\right)}^{T}P{\overset{˘}{B}}_{D}{\bar{\eta }}^{*}\left(k\right)\\ \; & +{\left(1-\bar{\beta }\right)}^{2}{\bar{\eta }}^{*T}\left(k\right){\overset{˘}{B}}_{D}^{T}P{\overset{˘}{B}}_{D}{\bar{\eta }}^{*}\left(k\right)+{\tilde{\alpha }}^{2}{\bar{\eta }}^{T}\left(k\right)\Delta {\overset{˘}{A}}_{1}^{T}P\Delta {\overset{˘}{A}}_{1}\bar{\eta }\left(k\right)+{\tilde{\beta }}^{2}{\bar{\eta }}^{T}\left(k\right){\bar{L}}_{C}^{T}P{\bar{L}}_{C}\bar{\eta }\left(k\right)\\ \; & +{\tilde{\beta }}^{2}{\bar{\eta }}^{T}\left(k\right){\overset{˘}{B}}_{L}^{T}P{\overset{˘}{B}}_{L}\bar{\eta }\left(k\right)-2{\tilde{\beta }}^{2}{\bar{\eta }}^{T}\left(k\right){\overset{˘}{B}}_{L}^{T}P{\overset{˘}{B}}_{D}{\bar{\eta }}^{*}\left(k\right)+w^{T}\left(k\right){\bar{G}}_{1}^{T}P{\bar{G}}_{1}w\left(k\right)\\ \; & +{\tilde{\beta }}^{2}{\bar{\eta }}^{*T}\left(k\right){\overset{˘}{B}}_{D}^{T}P{\overset{˘}{B}}_{D}{\bar{\eta }}^{*}\left(k\right)+f^{T}\left(k,I_{f}^{T}\bar{I}\bar{\eta }\left(k\right)\right){\bar{I}}_{f1}^{T}P{\bar{I}}_{f1}f\left(k,I_{f}^{T}\bar{I}\bar{\eta }\left(k\right)\right)\\ \; & +{\left(1-\bar{\beta }\right)}^{2}v^{T}\left(k\right){\bar{L}}_{D}^{T}P{\bar{L}}_{D}v\left(k\right)+{℘}_{1}{\left(1-\bar{\beta }\right)}^{2}v^{T}\left(k\right){\bar{L}}_{D}^{T}P{\bar{L}}_{D}v\left(k\right)+{℘}_{1}^{-1}{\left(1-\bar{\beta }\right)}^{2}{\tilde{q}}_{y}^{T}\left(k\right)\\ \; & \times {\bar{L}}_{1}^{T}P{\bar{L}}_{1}{\tilde{q}}_{y}\left(k\right)+{℘}_{2}{\left(1-\bar{\beta }\right)}^{2}v^{T}\left(k\right){\bar{L}}_{D}^{T}P{\bar{L}}_{D}v\left(k\right)+{℘}_{2}^{-1}{\left(1-\bar{\beta }\right)}^{2}{\tilde{r}}_{y}^{T}\left(k\right){\bar{L}}_{1}^{T}P{\bar{L}}_{1}{\tilde{r}}_{y}\left(k\right)\\ \; & +{\left(1-\bar{\beta }\right)}^{2}{\tilde{q}}_{y}^{T}\left(k\right){\bar{L}}_{1}^{T}P{\bar{L}}_{1}{\tilde{q}}_{y}\left(k\right)+{℘}_{3}{\left(1-\bar{\beta }\right)}^{2}{\tilde{q}}_{y}^{T}\left(k\right){\bar{L}}_{1}^{T}P{\bar{L}}_{1}{\tilde{q}}_{y}\left(k\right)\\ \; & +{℘}_{3}^{-1}{\left(1-\bar{\beta }\right)}^{2}{\tilde{r}}_{y}^{T}\left(k\right){\bar{L}}_{1}^{T}P{\bar{L}}_{1}{\tilde{r}}_{y}\left(k\right)+{\left(1-\bar{\beta }\right)}^{2}{\tilde{r}}_{y}^{T}\left(k\right){\bar{L}}_{1}^{T}P{\bar{L}}_{1}{\tilde{r}}_{y}\left(k\right)\\ \; & +{\tilde{\beta }}^{2}v^{T}\left(k\right){\bar{L}}_{D}^{T}P{\bar{L}}_{D}v\left(k\right)+{℘}_{4}{\tilde{\beta }}^{2}v^{T}\left(k\right){\bar{L}}_{D}^{T}P{\bar{L}}_{D}v\left(k\right)+{℘}_{4}^{-1}{\tilde{\beta }}^{2}{\tilde{q}}_{y}^{T}\left(k\right){\bar{L}}_{1}^{T}P{\bar{L}}_{1}{\tilde{q}}_{y}\left(k\right)\\ \; & +{℘}_{5}{\tilde{\beta }}^{2}v^{T}\left(k\right){\bar{L}}_{D}^{T}P{\bar{L}}_{D}v\left(k\right)+{℘}_{5}^{-1}{\tilde{\beta }}^{2}{\tilde{r}}_{y}^{T}\left(k\right){\bar{L}}_{1}^{T}P{\bar{L}}_{1}{\tilde{r}}_{y}\left(k\right)+{\tilde{\beta }}^{2}{\tilde{q}}_{y}^{T}\left(k\right){\bar{L}}_{1}^{T}P{\bar{L}}_{1}{\tilde{q}}_{y}\left(k\right)\\ \; & +{℘}_{6}{\tilde{\beta }}^{2}{\tilde{q}}_{y}^{T}\left(k\right){\bar{L}}_{1}^{T}P{\bar{L}}_{1}{\tilde{q}}_{y}\left(k\right)+{℘}_{6}^{-1}{\tilde{\beta }}^{2}{\tilde{r}}_{y}^{T}\left(k\right){\bar{L}}_{1}^{T}P{\bar{L}}_{1}{\tilde{r}}_{y}\left(k\right)+{\tilde{\beta }}^{2}{\tilde{r}}_{y}^{T}\left(k\right){\bar{L}}_{1}^{T}P{\bar{L}}_{1}{\tilde{r}}_{y}\left(k\right)\\ \; & +{\left(1-\bar{\beta }\right)}^{2}{\tilde{q}}_{\bar{u}}^{T}\left(k\right){\bar{B}}_{1}^{T}P{\bar{B}}_{1}{\tilde{q}}_{\bar{u}}\left(k\right)+{℘}_{7}{\left(1-\bar{\beta }\right)}^{2}{\tilde{q}}_{\bar{u}}^{T}\left(k\right){\bar{B}}_{1}^{T}P{\bar{B}}_{1}{\tilde{q}}_{\bar{u}}\left(k\right)+{℘}_{7}^{-1}{\left(1-\bar{\beta }\right)}^{2}{\tilde{r}}_{\bar{u}}^{T}\left(k\right){\bar{B}}_{1}^{T}P\\ \; & \times {\bar{B}}_{1}{\tilde{r}}_{\bar{u}}\left(k\right)+{\left(1-\bar{\beta }\right)}^{2}{\tilde{r}}_{\bar{u}}^{T}\left(k\right){\bar{B}}_{1}^{T}P{\bar{B}}_{1}{\tilde{r}}_{\bar{u}}\left(k\right)+{\tilde{\beta }}^{2}{\tilde{q}}_{\bar{u}}^{T}\left(k\right){\bar{B}}_{1}^{T}P{\bar{B}}_{1}{\tilde{q}}_{\bar{u}}\left(k\right)-{\bar{\eta }}^{T}\left(k\right)P\bar{\eta }\left(k\right)\\ \; & +{℘}_{8}{\tilde{\beta }}^{2}{\tilde{r}}_{\bar{u}}^{T}\left(k\right){\bar{B}}_{1}^{T}P{\bar{B}}_{1}{\tilde{r}}_{\bar{u}}\left(k\right)+{℘}_{8}^{-1}{\tilde{\beta }}^{2}{\tilde{q}}_{\bar{u}}^{T}\left(k\right){\bar{B}}_{1}^{T}P{\bar{B}}_{1}{\tilde{q}}_{\bar{u}}\left(k\right)+{\tilde{\beta }}^{2}{\tilde{r}}_{\bar{u}}^{T}\left(k\right){\bar{B}}_{1}^{T}P{\bar{B}}_{1}{\tilde{r}}_{\bar{u}}\left(k\right)\}\\ = & E\{{\bar{\eta }}^{T}\left(k\right){\left(\overset{˘}{A}+\bar{\alpha }\Delta {\overset{˘}{A}}_{1}\right)}^{T}P\left(\overset{˘}{A}+\bar{\alpha }\Delta {\overset{˘}{A}}_{1}\right)\bar{\eta }\left(k\right)+2\left(1-\bar{\beta }\right){\bar{\eta }}^{T}\left(k\right){\left(\overset{˘}{A}+\bar{\alpha }\Delta {\overset{˘}{A}}_{1}\right)}^{T}P{\overset{˘}{B}}_{D}{\bar{\eta }}^{*}\left(k\right)\\ \; & +{\left(1-\bar{\beta }\right)}^{2}{\bar{\eta }}^{*T}\left(k\right){\overset{˘}{B}}_{D}^{T}P{\overset{˘}{B}}_{D}{\bar{\eta }}^{*}\left(k\right)+{\tilde{\alpha }}^{2}{\bar{\eta }}^{T}\left(k\right)\Delta {\overset{˘}{A}}_{1}^{T}P\Delta {\overset{˘}{A}}_{1}\bar{\eta }\left(k\right)+{\tilde{\beta }}^{2}{\bar{\eta }}^{T}\left(k\right){\bar{L}}_{C}^{T}P{\bar{L}}_{C}\bar{\eta }\left(k\right)\\ \; & +{\tilde{\beta }}^{2}{\bar{\eta }}^{T}\left(k\right){\overset{˘}{B}}_{L}^{T}P{\overset{˘}{B}}_{L}\bar{\eta }\left(k\right)-2{\tilde{\beta }}^{2}{\bar{\eta }}^{T}\left(k\right){\overset{˘}{B}}_{L}^{T}P{\overset{˘}{B}}_{D}{\bar{\eta }}^{*}\left(k\right)+w^{T}\left(k\right){\bar{G}}_{1}^{T}P{\bar{G}}_{1}w\left(k\right)\\ \; & +{\tilde{\beta }}^{2}{\bar{\eta }}^{*T}\left(k\right){\overset{˘}{B}}_{D}^{T}P{\overset{˘}{B}}_{D}{\bar{\eta }}^{*}\left(k\right)+f^{T}\left(k,I_{f}^{T}\bar{I}\bar{\eta }\left(k\right)\right){\bar{I}}_{f1}^{T}P{\bar{I}}_{f1}f\left(k,I_{f}^{T}\bar{I}\bar{\eta }\left(k\right)\right)\\ \; & +\left({\left(1-\bar{\beta }\right)}^{2}+{℘}_{1}{\left(1-\bar{\beta }\right)}^{2}+{℘}_{2}{\left(1-\bar{\beta }\right)}^{2}+{\tilde{\beta }}^{2}+{℘}_{4}{\tilde{\beta }}^{2}+{℘}_{5}{\tilde{\beta }}^{2}\right)v^{T}\left(k\right){\bar{L}}_{D}^{T}P{\bar{L}}_{D}v\left(k\right)\\ \; & +\left({℘}_{1}^{-1}{\left(1-\bar{\beta }\right)}^{2}+{\left(1-\bar{\beta }\right)}^{2}+{℘}_{3}{\left(1-\bar{\beta }\right)}^{2}+{℘}_{4}^{-1}{\tilde{\beta }}^{2}+{\tilde{\beta }}^{2}+{℘}_{6}{\tilde{\beta }}^{2}\right){\tilde{q}}_{y}^{T}\left(k\right){\bar{L}}_{1}^{T}P{\bar{L}}_{1}{\tilde{q}}_{y}\left(k\right)\\ \; & +\left({℘}_{2}^{-1}{\left(1-\bar{\beta }\right)}^{2}+{℘}_{3}^{-1}{\left(1-\bar{\beta }\right)}^{2}+{\left(1-\bar{\beta }\right)}^{2}+{℘}_{5}^{-1}{\tilde{\beta }}^{2}+{℘}_{6}^{-1}{\tilde{\beta }}^{2}+{\tilde{\beta }}^{2}\right){\tilde{r}}_{y}^{T}\left(k\right){\bar{L}}_{1}^{T}P{\bar{L}}_{1}{\tilde{r}}_{y}\left(k\right)\\ \; & +\left({\left(1-\bar{\beta }\right)}^{2}+{℘}_{7}{\left(1-\bar{\beta }\right)}^{2}+{℘}_{8}^{-1}{\tilde{\beta }}^{2}+{\tilde{\beta }}^{2}\right){\tilde{q}}_{\bar{u}}^{T}\left(k\right){\bar{B}}_{1}^{T}P{\bar{B}}_{1}{\tilde{q}}_{\bar{u}}\left(k\right)\\ \; & +\left({℘}_{7}^{-1}{\left(1-\bar{\beta }\right)}^{2}+{\left(1-\bar{\beta }\right)}^{2}+{℘}_{8}{\tilde{\beta }}^{2}+{\tilde{\beta }}^{2}\right){\tilde{r}}_{\bar{u}}^{T}\left(k\right){\bar{B}}_{1}^{T}P{\bar{B}}_{1}{\tilde{r}}_{\bar{u}}\left(k\right)-{\bar{\eta }}^{T}\left(k\right)P\bar{\eta }\left(k\right)\}\\ = & E\{{\bar{\eta }}^{T}\left(k\right){\left(\overset{˘}{A}+\bar{\alpha }\Delta {\overset{˘}{A}}_{1}\right)}^{T}P\left(\overset{˘}{A}+\bar{\alpha }\Delta {\overset{˘}{A}}_{1}\right)\bar{\eta }\left(k\right)+2\left(1-\bar{\beta }\right){\bar{\eta }}^{T}\left(k\right){\left(\overset{˘}{A}+\bar{\alpha }\Delta {\overset{˘}{A}}_{1}\right)}^{T}P{\overset{˘}{B}}_{D}{\bar{\eta }}^{*}\left(k\right)\\ \; & +{\left(1-\bar{\beta }\right)}^{2}{\bar{\eta }}^{*T}\left(k\right){\overset{˘}{B}}_{D}^{T}P{\overset{˘}{B}}_{D}{\bar{\eta }}^{*}\left(k\right)+{\tilde{\alpha }}^{2}{\bar{\eta }}^{T}\left(k\right)\Delta {\overset{˘}{A}}_{1}^{T}P\Delta {\overset{˘}{A}}_{1}\bar{\eta }\left(k\right)+{\tilde{\beta }}^{2}{\bar{\eta }}^{T}\left(k\right){\bar{L}}_{C}^{T}P{\bar{L}}_{C}\bar{\eta }\left(k\right)\\ \; & +{\tilde{\beta }}^{2}{\bar{\eta }}^{T}\left(k\right){\overset{˘}{B}}_{L}^{T}P{\overset{˘}{B}}_{L}\bar{\eta }\left(k\right)-2{\tilde{\beta }}^{2}{\bar{\eta }}^{T}\left(k\right){\overset{˘}{B}}_{L}^{T}P{\overset{˘}{B}}_{D}{\bar{\eta }}^{*}\left(k\right)+w^{T}\left(k\right){\bar{G}}_{1}^{T}P{\bar{G}}_{1}w\left(k\right)\\ \; & +{\tilde{\beta }}^{2}{\bar{\eta }}^{*T}\left(k\right){\overset{˘}{B}}_{D}^{T}P{\overset{˘}{B}}_{D}{\bar{\eta }}^{*}\left(k\right)+f^{T}\left(k,I_{f}^{T}\bar{I}\bar{\eta }\left(k\right)\right){\bar{I}}_{f1}^{T}P{\bar{I}}_{f1}f\left(k,I_{f}^{T}\bar{I}\bar{\eta }\left(k\right)\right)\\ \; & +{℧}_{1}v^{T}\left(k\right){\bar{L}}_{D}^{T}P{\bar{L}}_{D}v\left(k\right)+{℧}_{2}{\tilde{q}}_{y}^{T}\left(k\right){\bar{L}}_{1}^{T}P{\bar{L}}_{1}{\tilde{q}}_{y}\left(k\right)+{℧}_{3}{\tilde{r}}_{y}^{T}\left(k\right){\bar{L}}_{1}^{T}P{\bar{L}}_{1}{\tilde{r}}_{y}\left(k\right)\\ \; & +{℧}_{4}{\tilde{q}}_{\bar{u}}^{T}\left(k\right){\bar{B}}_{1}^{T}P{\bar{B}}_{1}{\tilde{q}}_{\bar{u}}\left(k\right)+{℧}_{5}{\tilde{r}}_{\bar{u}}^{T}\left(k\right){\bar{B}}_{1}^{T}P{\bar{B}}_{1}{\tilde{r}}_{\bar{u}}\left(k\right)-{\bar{\eta }}^{T}\left(k\right)P\bar{\eta }\left(k\right)\} \end{array}$$

where

$$\begin{array}{cc} {℧}_{1}≜ & {\left(1-\bar{\beta }\right)}^{2}+{℘}_{1}{\left(1-\bar{\beta }\right)}^{2}+{℘}_{2}{\left(1-\bar{\beta }\right)}^{2}+{\tilde{\beta }}^{2}+{℘}_{4}{\tilde{\beta }}^{2}+{℘}_{5}{\tilde{\beta }}^{2},\\ {℧}_{2}≜ & {℘}_{1}^{-1}{\left(1-\bar{\beta }\right)}^{2}+{\left(1-\bar{\beta }\right)}^{2}+{℘}_{3}{\left(1-\bar{\beta }\right)}^{2}+{℘}_{4}^{-1}{\tilde{\beta }}^{2}+{\tilde{\beta }}^{2}+{℘}_{6}{\tilde{\beta }}^{2},\\ {℧}_{3}≜ & {℘}_{2}^{-1}{\left(1-\bar{\beta }\right)}^{2}+{℘}_{3}^{-1}{\left(1-\bar{\beta }\right)}^{2}+{\left(1-\bar{\beta }\right)}^{2}+{℘}_{5}^{-1}{\tilde{\beta }}^{2}+{℘}_{6}^{-1}{\tilde{\beta }}^{2}+{\tilde{\beta }}^{2},\\ {℧}_{4}≜ & {\left(1-\bar{\beta }\right)}^{2}+{℘}_{7}{\left(1-\bar{\beta }\right)}^{2}+{℘}_{8}^{-1}{\tilde{\beta }}^{2}+{\tilde{\beta }}^{2},\\ {℧}_{5}≜ & {℘}_{7}^{-1}{\left(1-\bar{\beta }\right)}^{2}+{\left(1-\bar{\beta }\right)}^{2}+{℘}_{8}{\tilde{\beta }}^{2}+{\tilde{\beta }}^{2}. \end{array}$$

On the basis of the variance properties (22) and (27), the properties of matrix trace including $\mathrm{tr}\left(c_{1}\right)=c_{1}$ (where $c_{1}$ is an arbitrary scalar), $\mathrm{tr}\left(A_{1}B_{1}\right)=\mathrm{tr}\left(B_{1}A_{1}\right)$ and $\mathrm{tr}\left(\mathrm{diag}\left\{A_{1},B_{1}\right\}\right)=\mathrm{tr}\left(A_{1}\right)+\mathrm{tr}\left(B_{1}\right)$ (where $A_{1}$ and $B_{1}$ are arbitrary square matrices), the following inequality is derived:

(40) $$\begin{array}{cc} \; & E\{\Delta V_{1}\left(k\right)\}\\ \leq & E\{{\bar{\eta }}^{T}\left(k\right){\left(\overset{˘}{A}+\bar{\alpha }\Delta {\overset{˘}{A}}_{1}\right)}^{T}P\left(\overset{˘}{A}+\bar{\alpha }\Delta {\overset{˘}{A}}_{1}\right)\bar{\eta }\left(k\right)+2\left(1-\bar{\beta }\right){\bar{\eta }}^{T}\left(k\right){\left(\overset{˘}{A}+\bar{\alpha }\Delta {\overset{˘}{A}}_{1}\right)}^{T}P{\overset{˘}{B}}_{D}{\bar{\eta }}^{*}\left(k\right)\\ \; & +{\left(1-\bar{\beta }\right)}^{2}{\bar{\eta }}^{*T}\left(k\right){\overset{˘}{B}}_{D}^{T}P{\overset{˘}{B}}_{D}{\bar{\eta }}^{*}\left(k\right)+{\tilde{\alpha }}^{2}{\bar{\eta }}^{T}\left(k\right)\Delta {\overset{˘}{A}}_{1}^{T}P\Delta {\overset{˘}{A}}_{1}\bar{\eta }\left(k\right)+{\tilde{\beta }}^{2}{\bar{\eta }}^{T}\left(k\right){\bar{L}}_{C}^{T}P{\bar{L}}_{C}\bar{\eta }\left(k\right)\\ \; & +{\tilde{\beta }}^{2}{\bar{\eta }}^{T}\left(k\right){\overset{˘}{B}}_{L}^{T}P{\overset{˘}{B}}_{L}\bar{\eta }\left(k\right)-2{\tilde{\beta }}^{2}{\bar{\eta }}^{T}\left(k\right){\overset{˘}{B}}_{L}^{T}P{\overset{˘}{B}}_{D}{\bar{\eta }}^{*}\left(k\right)-{\bar{\eta }}^{T}\left(k\right)P\bar{\eta }\left(k\right)\\ \; & +{\tilde{\beta }}^{2}{\bar{\eta }}^{*T}\left(k\right){\overset{˘}{B}}_{D}^{T}P{\overset{˘}{B}}_{D}{\bar{\eta }}^{*}\left(k\right)+\mathrm{tr}\left({\bar{I}}_{f1}^{T}P{\bar{I}}_{f1}f\left(k,I_{f}^{T}\bar{I}\bar{\eta }\left(k\right)\right)f^{T}\left(k,I_{f}^{T}\bar{I}\bar{\eta }\left(k\right)\right)\right)\\ \; & +\mathrm{tr}\left({\bar{G}}_{1}^{T}P{\bar{G}}_{1}w\left(k\right)w^{T}\left(k\right)\right)+\mathrm{tr}\left({℧}_{1}{\bar{L}}_{D}^{T}P{\bar{L}}_{D}v\left(k\right)v^{T}\left(k\right)\right)+\mathrm{tr}\left({℧}_{2}{\bar{L}}_{1}^{T}P{\bar{L}}_{1}{\tilde{q}}_{y}\left(k\right){\tilde{q}}_{y}^{T}\left(k\right)\right)\\ \; & +\mathrm{tr}\left({℧}_{3}{\bar{L}}_{1}^{T}P{\bar{L}}_{1}{\tilde{r}}_{y}\left(k\right){\tilde{r}}_{y}^{T}\left(k\right)\right)+\mathrm{tr}\left({℧}_{4}{\bar{B}}_{1}^{T}P{\bar{B}}_{1}{\tilde{q}}_{\bar{u}}\left(k\right){\tilde{q}}_{\bar{u}}^{T}\left(k\right)\right)\\ \; & +\mathrm{tr}({℧}_{5}{\bar{B}}_{1}^{T}P{\bar{B}}_{1}{\tilde{r}}_{\bar{u}}\left(k\right){\tilde{r}}_{\bar{u}}^{T}\left(k\right))\}\\ \leq & E\{{\bar{\eta }}^{T}\left(k\right){\left(\overset{˘}{A}+\bar{\alpha }\Delta {\overset{˘}{A}}_{1}\right)}^{T}P\left(\overset{˘}{A}+\bar{\alpha }\Delta {\overset{˘}{A}}_{1}\right)\bar{\eta }\left(k\right)+2\left(1-\bar{\beta }\right){\bar{\eta }}^{T}\left(k\right){\left(\overset{˘}{A}+\bar{\alpha }\Delta {\overset{˘}{A}}_{1}\right)}^{T}P{\overset{˘}{B}}_{D}{\bar{\eta }}^{*}\left(k\right)\\ \; & +{\left(1-\bar{\beta }\right)}^{2}{\bar{\eta }}^{*T}\left(k\right){\overset{˘}{B}}_{D}^{T}P{\overset{˘}{B}}_{D}{\bar{\eta }}^{*}\left(k\right)+{\tilde{\alpha }}^{2}{\bar{\eta }}^{T}\left(k\right)\Delta {\overset{˘}{A}}_{1}^{T}P\Delta {\overset{˘}{A}}_{1}\bar{\eta }\left(k\right)+{\tilde{\beta }}^{2}{\bar{\eta }}^{T}\left(k\right){\bar{L}}_{C}^{T}P{\bar{L}}_{C}\bar{\eta }\left(k\right)\\ \; & +{\tilde{\beta }}^{2}{\bar{\eta }}^{T}\left(k\right){\overset{˘}{B}}_{L}^{T}P{\overset{˘}{B}}_{L}\bar{\eta }\left(k\right)-2{\tilde{\beta }}^{2}{\bar{\eta }}^{T}\left(k\right){\overset{˘}{B}}_{L}^{T}P{\overset{˘}{B}}_{D}{\bar{\eta }}^{*}\left(k\right)\\ \; & +{\tilde{\beta }}^{2}{\bar{\eta }}^{*T}\left(k\right){\overset{˘}{B}}_{D}^{T}P{\overset{˘}{B}}_{D}{\bar{\eta }}^{*}\left(k\right)+\mathrm{tr}\left({\bar{I}}_{f1}^{T}P{\bar{I}}_{f1}f\left(k,I_{f}^{T}\bar{I}\bar{\eta }\left(k\right)\right)f^{T}\left(k,I_{f}^{T}\bar{I}\bar{\eta }\left(k\right)\right)\right)\\ \; & +\mathrm{tr}\left(w_{0}{\bar{G}}_{1}^{T}P{\bar{G}}_{1}\right)+\mathrm{tr}\left({℧}_{1}v_{0}{\bar{L}}_{D}^{T}P{\bar{L}}_{D}\right)+\mathrm{tr}\left({℧}_{2}\left(\theta^{2}/4\right){\bar{L}}_{1}^{T}P{\bar{L}}_{1}\right)\\ \; & +\mathrm{tr}\left({℧}_{3}\left(1/{\left(1-2\bar{϶}\right)}^{2}\right){ℑ}^{2}\pi {\bar{L}}_{1}^{T}P{\bar{L}}_{1}\right)+\mathrm{tr}\left({℧}_{4}\left(\theta^{2}/4\right){\bar{B}}_{1}^{T}P{\bar{B}}_{1}\right)\\ \; & +\mathrm{tr}\left({℧}_{5}\left(1/{\left(1-2\bar{϶}\right)}^{2}\right){ℑ}^{2}\pi {\bar{B}}_{1}^{T}P{\bar{B}}_{1}\right)-{\bar{\eta }}^{T}\left(k\right)P\bar{\eta }\left(k\right)\}\\ = & E\{{\bar{\eta }}^{T}\left(k\right){\left(\overset{˘}{A}+\bar{\alpha }\Delta {\overset{˘}{A}}_{1}\right)}^{T}P\left(\overset{˘}{A}+\bar{\alpha }\Delta {\overset{˘}{A}}_{1}\right)\bar{\eta }\left(k\right)+2\left(1-\bar{\beta }\right){\bar{\eta }}^{T}\left(k\right){\left(\overset{˘}{A}+\bar{\alpha }\Delta {\overset{˘}{A}}_{1}\right)}^{T}P{\overset{˘}{B}}_{D}{\bar{\eta }}^{*}\left(k\right)\\ \; & +{\left(1-\bar{\beta }\right)}^{2}{\bar{\eta }}^{*T}\left(k\right){\overset{˘}{B}}_{D}^{T}P{\overset{˘}{B}}_{D}{\bar{\eta }}^{*}\left(k\right)+{\tilde{\alpha }}^{2}{\bar{\eta }}^{T}\left(k\right)\Delta {\overset{˘}{A}}_{1}^{T}P\Delta {\overset{˘}{A}}_{1}\bar{\eta }\left(k\right)+{\tilde{\beta }}^{2}{\bar{\eta }}^{T}\left(k\right){\bar{L}}_{C}^{T}P{\bar{L}}_{C}\bar{\eta }\left(k\right)\\ \; & +{\tilde{\beta }}^{2}{\bar{\eta }}^{T}\left(k\right){\overset{˘}{B}}_{L}^{T}P{\overset{˘}{B}}_{L}\bar{\eta }\left(k\right)-2{\tilde{\beta }}^{2}{\bar{\eta }}^{T}\left(k\right){\overset{˘}{B}}_{L}^{T}P{\overset{˘}{B}}_{D}{\bar{\eta }}^{*}\left(k\right)-{\bar{\eta }}^{T}\left(k\right)P\bar{\eta }\left(k\right)\\ \; & +{\tilde{\beta }}^{2}{\bar{\eta }}^{*T}\left(k\right){\overset{˘}{B}}_{D}^{T}P{\overset{˘}{B}}_{D}{\bar{\eta }}^{*}\left(k\right)+\mathrm{tr}\left({\bar{I}}_{f1}^{T}P{\bar{I}}_{f1}f\left(k,I_{f}^{T}\bar{I}\bar{\eta }\left(k\right)\right)f^{T}\left(k,I_{f}^{T}\bar{I}\bar{\eta }\left(k\right)\right)\right)+\mathrm{tr}\left(\Upsilon \right)\} \end{array}$$

where

$$\begin{array}{cc} \Upsilon ≜\mathrm{diag}\{\; & w_{0}{\bar{G}}_{1}^{T}P{\bar{G}}_{1},{℧}_{1}v_{0}{\bar{L}}_{D}^{T}P{\bar{L}}_{D},\\ \; & {℧}_{2}\left(\theta^{2}/4\right){\bar{L}}_{1}^{T}P{\bar{L}}_{1}+{℧}_{3}\left(1/{\left(1-2\bar{϶}\right)}^{2}\right){ℑ}^{2}\pi {\bar{L}}_{1}^{T}P{\bar{L}}_{1},\\ \; & {℧}_{4}\left(\theta^{2}/4\right){\bar{B}}_{1}^{T}P{\bar{B}}_{1}+{℧}_{5}\left(1/{\left(1-2\bar{϶}\right)}^{2}\right){ℑ}^{2}\pi {\bar{B}}_{1}^{T}P{\bar{B}}_{1}\}. \end{array}$$

On the basis of (2), we have

(41) $$\begin{array}{cc} \; & E\{\mathrm{tr}\left({\bar{I}}_{f1}^{T}P{\bar{I}}_{f1}f\left(k,I_{f}^{T}\bar{I}\bar{\eta }\left(k\right)\right)f^{T}\left(k,I_{f}^{T}\bar{I}\bar{\eta }\left(k\right)\right)\right)\}\\ = & E\{\mathrm{tr}\left({\bar{I}}_{f1}^{T}P{\bar{I}}_{f1}\sum_{l=1}^{\xi }\Theta_{l}{\left(I_{f}^{T}\bar{I}\bar{\eta }\left(k\right)\right)}^{T}\Gamma_{l}I_{f}^{T}\bar{I}\bar{\eta }\left(k\right)\right)\}\\ = & E\{\mathrm{tr}\left({\bar{I}}_{f1}^{T}P{\bar{I}}_{f1}\sum_{l=1}^{\xi }\Theta_{l}{\bar{\eta }}^{T}\left(k\right){\bar{I}}^{T}I_{f}\Gamma_{l}I_{f}^{T}\bar{I}\bar{\eta }\left(k\right)\right)\}\\ = & E\{\mathrm{tr}\left({\bar{I}}_{f1}^{T}P{\bar{I}}_{f1}\sum_{l=1}^{\xi }\Theta_{l}\right){\bar{\eta }}^{T}\left(k\right){\bar{I}}^{T}I_{f}\Gamma_{l}I_{f}^{T}\bar{I}\bar{\eta }\left(k\right)\}\\ = & E\{\sum_{l=1}^{\xi }\mathrm{tr}\left({\bar{I}}_{f1}^{T}P{\bar{I}}_{f1}\Theta_{l}\right){\bar{\eta }}^{T}\left(k\right){\bar{I}}^{T}I_{f}\Gamma_{l}I_{f}^{T}\bar{I}\bar{\eta }\left(k\right)\}\\ = & E\{{\bar{\eta }}^{T}\left(k\right)\sum_{l=1}^{\xi }\mathrm{tr}\left({\bar{I}}_{f1}^{T}P{\bar{I}}_{f1}\Theta_{l}\right){\bar{I}}^{T}I_{f}\Gamma_{l}I_{f}^{T}\bar{I}\bar{\eta }\left(k\right)\}\\ = & E\{{\bar{\eta }}^{T}\left(k\right)\sum_{l=1}^{\xi }\mathrm{tr}\left({\bar{I}}_{f1}^{T}P{\bar{I}}_{f1}\iota_{l}\iota_{l}^{T}\right){\bar{I}}^{T}I_{f}\Gamma_{l}I_{f}^{T}\bar{I}\bar{\eta }\left(k\right)\}\\ = & E\{{\bar{\eta }}^{T}\left(k\right)\sum_{l=1}^{\xi }\mathrm{tr}\left(\iota_{l}^{T}{\bar{I}}_{f1}^{T}P{\bar{I}}_{f1}\iota_{l}\right){\bar{I}}^{T}I_{f}\Gamma_{l}I_{f}^{T}\bar{I}\bar{\eta }\left(k\right)\}. \end{array}$$

Combining (40) and (41), one attains

(42) $$\begin{array}{cc} \; & E\{\Delta V_{1}\left(k\right)\}\\ \leq & E\{{\bar{\eta }}^{T}\left(k\right){\left(\overset{˘}{A}+\bar{\alpha }\Delta {\overset{˘}{A}}_{1}\right)}^{T}P\left(\overset{˘}{A}+\bar{\alpha }\Delta {\overset{˘}{A}}_{1}\right)\bar{\eta }\left(k\right)+2\left(1-\bar{\beta }\right){\bar{\eta }}^{T}\left(k\right){\left(\overset{˘}{A}+\bar{\alpha }\Delta {\overset{˘}{A}}_{1}\right)}^{T}P{\overset{˘}{B}}_{D}{\bar{\eta }}^{*}\left(k\right)\\ \; & +{\left(1-\bar{\beta }\right)}^{2}{\bar{\eta }}^{*T}\left(k\right){\overset{˘}{B}}_{D}^{T}P{\overset{˘}{B}}_{D}{\bar{\eta }}^{*}\left(k\right)+{\tilde{\alpha }}^{2}{\bar{\eta }}^{T}\left(k\right)\Delta {\overset{˘}{A}}_{1}^{T}P\Delta {\overset{˘}{A}}_{1}\bar{\eta }\left(k\right)+{\tilde{\beta }}^{2}{\bar{\eta }}^{T}\left(k\right){\bar{L}}_{C}^{T}P{\bar{L}}_{C}\bar{\eta }\left(k\right)\\ \; & +{\tilde{\beta }}^{2}{\bar{\eta }}^{T}\left(k\right){\overset{˘}{B}}_{L}^{T}P{\overset{˘}{B}}_{L}\bar{\eta }\left(k\right)-2{\tilde{\beta }}^{2}{\bar{\eta }}^{T}\left(k\right){\overset{˘}{B}}_{L}^{T}P{\overset{˘}{B}}_{D}{\bar{\eta }}^{*}\left(k\right)-{\bar{\eta }}^{T}\left(k\right)P\bar{\eta }\left(k\right)\\ \; & +{\tilde{\beta }}^{2}{\bar{\eta }}^{*T}\left(k\right){\overset{˘}{B}}_{D}^{T}P{\overset{˘}{B}}_{D}{\bar{\eta }}^{*}\left(k\right)+{\bar{\eta }}^{T}\left(k\right)\sum_{l=1}^{\xi }\mathrm{tr}\left(\iota_{l}^{T}{\bar{I}}_{f1}^{T}P{\bar{I}}_{f1}\iota_{l}\right){\bar{I}}^{T}I_{f}\Gamma_{l}I_{f}^{T}\bar{I}\bar{\eta }\left(k\right)+\mathrm{tr}\left(\Upsilon \right)\}. \end{array}$$

In terms of (37) and (42), we get the following inequality:

(43) $$\begin{array}{cc} E\{\Delta V(k)\}= & E\left\{\Delta V_{1}\left(k\right)\right\}+E\left\{\Delta V_{2}\left(k\right)\right\}\leq E\left\{{\bar{X}}^{T}\left(k\right)\Pi \bar{X}\left(k\right)\right\}+\mathrm{tr}\left(\Upsilon \right) \end{array}$$

where $\bar{X}\left(k\right)≜{\left[\begin{array}{cc} {\bar{\eta }}^{T}\left(k\right) & {\bar{\eta }}^{*T}\left(k\right) \end{array}\right]}^{T}$.

Considering (34), there exists a scalar δ>0 satisfying

(44) $$\begin{array}{c} E\left\{\Delta V\left(k\right)\right\}\leq -\delta \underset{i\leq k}{\sup}E\left\{∥\bar{\eta }{\left(i\right)∥}^{2}\right\}+\mathrm{tr}\left(\Upsilon \right). \end{array}$$

Based on (35), we acquire that

(45) $$\begin{array}{c} E\left\{V\left(k\right)\right\}\leq \rho \underset{i\leq k}{\sup}E\left\{∥\bar{\eta }{\left(i\right)∥}^{2}\right\} \end{array}$$

where $\rho ≜\lambda_{\max}\left(P\right)+N\lambda_{\max}\left(\sum_{j=1}^{N}Q_{j}\right)$.

When k=0, it is obtained from (45) that

(46) $$\begin{array}{c} E\left\{V\left(0\right)\right\}\leq \rho \underset{i\leq 0}{\sup}E\left\{∥\bar{\eta }{\left(i\right)∥}^{2}\right\}. \end{array}$$

Concerning a scalar h>1 and inequalities (44) and (45), we yield the following inequality:

(47) $$\begin{array}{cc} \; & h^{k+1}E\left\{V\left(k+1\right)\right\}-h^{k}E\left\{V\left(k\right)\right\}\\ = & h^{k+1}E\left\{V\left(k+1\right)\right\}-h^{k+1}E\left\{V\left(k\right)\right\}+h^{k+1}E\left\{V\left(k\right)\right\}-h^{k}E\left\{V\left(k\right)\right\}\\ = & h^{k+1}E\left\{\Delta V\left(k\right)\right\}+h^{k}\left(h-1\right)E\left\{V\left(k\right)\right\}\\ \leq & h^{k+1}\left(-\delta \underset{i\leq k}{\sup}E\left\{∥\bar{\eta }{\left(i\right)∥}^{2}\right\}+\mathrm{tr}\left(\Upsilon \right)\right)+h^{k}\left(h-1\right)\left(\rho \underset{i\leq k}{\sup}E\left\{∥\bar{\eta }{\left(i\right)∥}^{2}\right\}\right)\\ = & h^{k}\left(-h\delta +\left(h-1\right)\rho \right)\underset{i\leq k}{\sup}E\left\{∥\bar{\eta }{\left(i\right)∥}^{2}\right\}+h^{k+1}\mathrm{tr}\left(\Upsilon \right)\\ = & h^{k}\varpi \left(h\right)\underset{i\leq k}{\sup}E\left\{∥\bar{\eta }{\left(i\right)∥}^{2}\right\}+h^{k+1}\mathrm{tr}\left(\Upsilon \right) \end{array}$$

where ϖ(h)≜−hδ+(h−1)ρ.

Summing on both sides of (47) from k=0 to k=Ω, one gets

(48) $$\begin{array}{c} h^{\Omega +1}E\left\{V\left(\Omega +1\right)\right\}-E\left\{V\left(0\right)\right\}\leq \varpi \left(h\right)\sum_{k=0}^{\Omega }h^{k}\underset{i\leq k}{\sup}E\{∥\bar{\eta }\left(i\right){∥}^{2}\}+\frac{h(1-h^{\Omega +1})}{1-h}\mathrm{tr}\left(\Upsilon \right), \end{array}$$

that is,

(49) $$\begin{array}{c} h^{\Omega +1}E\left\{V\left(\Omega +1\right)\right\}\leq E\left\{V\left(0\right)\right\}+\varpi \left(h\right)\sum_{k=0}^{\Omega }h^{k}\underset{i\leq k}{\sup}E\{∥\bar{\eta }\left(i\right){∥}^{2}\}+\frac{h(1-h^{\Omega +1})}{1-h}\mathrm{tr}\left(\Upsilon \right). \end{array}$$

It is readily to know that there is a scalar $h_{0}>1$ such that $\varpi \left(h_{0}\right)=-h_{0}\delta +\left(h_{0}-1\right)\rho =0$. Then, the following expression is obtained from (49):

(50) $$\begin{array}{cc} E\{V(\Omega +1)\}\leq & \frac{E\{V(0)\}}{h_{0}^{\Omega +1}}+\frac{h_{0}\left(h_{0}^{-(\Omega +1)}-1\right)}{1-h_{0}}\mathrm{tr}\left(\Upsilon \right)\\ = & \frac{E\{V(0)\}}{h_{0}^{\Omega +1}}+\frac{h_{0}\left(1-h_{0}^{-(\Omega +1)}\right)}{h_{0}-1}\mathrm{tr}\left(\Upsilon \right)\\ < & \frac{E\{V(0)\}}{h_{0}^{\Omega +1}}+\frac{h_{0}}{h_{0}-1}\mathrm{tr}\left(\Upsilon \right). \end{array}$$

For (35), we see that the following formula holds:

(51) $$\begin{array}{cc} E\{V(k+1)\}\geq & E\left\{V_{2}\left(k+1\right)\right\}=E\left\{\sum_{j=1}^{N}\sum_{i=k-j+1}^{k}{\bar{\eta }}^{T}\left(i\right)Q_{j}\bar{\eta }\left(i\right)\right\}\\ \geq & E\left\{\sum_{j=1}^{N}{\bar{\eta }}^{T}\left(k\right)Q_{j}\bar{\eta }\left(k\right)\right\}=E\left\{{\bar{\eta }}^{T}\left(k\right)\sum_{j=1}^{N}Q_{j}\bar{\eta }\left(k\right)\right\}\\ \geq & E\left\{\lambda_{\min}\left(\sum_{j=1}^{N}Q_{j}\right){∥\bar{\eta }\left(k\right)∥}^{2}\right\}=E\{\chi ∥\bar{\eta }\left(k\right){∥}^{2}\} \end{array}$$

where $\chi ≜\lambda_{\min}\left(\sum_{j=1}^{N}Q_{j}\right)$.

From (51), we derive that $E\left\{V\left(\Omega +1\right)\right\}\geq \chi E\{∥\bar{\eta }\left(\Omega \right){∥}^{2}\}$. Furthermore, the inequality below is attained based on (46) and (50):

(52) $$\begin{array}{cc} E\{∥\bar{\eta }\left(\Omega \right){∥}^{2}\}\leq & \frac{E\{V(\Omega +1)\}}{\chi }\leq \frac{E\{V(0)\}}{\chi h_{0}^{\Omega +1}}+\frac{h_{0}}{\chi (h_{0}-1)}\mathrm{tr}\left(\Upsilon \right)\\ \leq & \frac{\rho \underset{i\leq 0}{\sup}E\left\{∥\bar{\eta }{\left(i\right)∥}^{2}\right\}}{\chi h_{0}^{\Omega +1}}+\frac{h_{0}}{\chi (h_{0}-1)}\mathrm{tr}\left(\Upsilon \right)\\ = & c_{0}(\frac{1}{h_{0}}{)}^{\Omega +1}\underset{i\leq 0}{\sup}E\{∥\bar{\eta }\left(i\right){∥}^{2}\}+\frac{h_{0}}{\chi (h_{0}-1)}\mathrm{tr}\left(\Upsilon \right) \end{array}$$

where $c_{0}≜\frac{\rho }{\chi }$.

According to (32), system (31) realizes EUBMS performance, and the ultimate bound is $\frac{h_{0}}{\chi (h_{0}-1)}\mathrm{tr}\left(\Upsilon \right)$. □

**Theorem** **2.** Given scalars $h_{0}>1$ and ${℘}_{𝚥}>0\;$(𝚥=1,2,…,8), the closed-loop system (31) subject to uncertainty, nonlinearity, integral measurements and DoS attacks using a BES and PID controller achieves EUBMS performance, if there exist matrices ${\bar{M}}_{1}$, ${\bar{M}}_{2}$, ${\bar{M}}_{3}$, ${\hat{L}}_{1}≜\left[\begin{array}{c} 0\\ \overset{˘}{L} \end{array}\right]$, ${\overset{˘}{K}}_{P}≜\left[\begin{array}{c} {\bar{K}}_{P}\\ 0 \end{array}\right]$, ${\overset{˘}{K}}_{I}≜\left[\begin{array}{c} {\bar{K}}_{I}\\ 0 \end{array}\right]$, ${\overset{˘}{K}}_{D}≜\left[\begin{array}{c} {\bar{K}}_{D}\\ 0 \end{array}\right]$, $P≜\mathrm{diag}\{P_{1},P_{2}\}>0$ and $Q_{j}≜\left[\begin{array}{cc} Q_{11j} & *\\ Q_{21j} & Q_{22j} \end{array}\right]>0\;$(j=1,2,…,N), $\overset{˘}{\Upsilon }>0$ and scalars ν>0 and ζ>0 such that the following inequalities hold:

(53) $$\begin{array}{cc} \; & \left[\begin{array}{cc} -\nu & *\\ P{\bar{I}}_{f1}\iota_{l} & -P \end{array}\right]<0 \end{array}$$

(54) $$\begin{array}{cc} \; & \left[\begin{array}{cccc} \Psi_{0} & * & * & *\\ {\tilde{\Psi }}_{B1} & P_{\bar{M}} & * & *\\ 0 & E_{1}^{T}\left(I_{\left(4\right)}⊗{\tilde{M}}^{T}\right) & -\zeta I & *\\ \zeta N_{1} & 0 & 0 & -\zeta I \end{array}\right]<0 \end{array}$$

(55) $$\begin{array}{cc} \; & {\tilde{H}}^{T}\tilde{H}<P \end{array}$$

(56) $$\begin{array}{cc} \; & \left[\begin{array}{ccc} -\overset{˘}{\Upsilon } & * & *\\ {\overset{˘}{\Upsilon }}_{M21} & -I_{\left(4\right)}⊗P & *\\ {\overset{˘}{\Upsilon }}_{M31} & 0 & -I_{\left(4\right)}⊗P \end{array}\right]<0 \end{array}$$

*where*

$$\begin{array}{cc} \; & \bar{W}≜{\left[\begin{array}{cc} \bar{B}{\left({\bar{B}}^{T}\bar{B}\right)}^{-1} & {\left({\bar{B}}^{T}\right)}^{⊥} \end{array}\right]}^{T},\bar{M}≜\left[\begin{array}{cc} {\bar{M}}_{1} & {\bar{M}}_{3}\\ 0 & {\bar{M}}_{2} \end{array}\right],E_{1}≜\left[\begin{array}{c} \bar{\alpha }{\overset{˘}{E}}_{1}\\ \tilde{\alpha }{\overset{˘}{E}}_{1}\\ 0\\ 0 \end{array}\right],{\overset{˘}{N}}_{1}≜\left[\begin{array}{cc} N_{1} & 0\\ N_{1} & 0 \end{array}\right],\\ \; & \Psi_{0}≜\mathrm{diag}\left\{\Psi_{01},-Q\right\},\Psi_{01}≜-P+\sum_{j=1}^{N}Q_{j}+\sum_{l=1}^{\xi }\nu {\bar{I}}^{T}I_{f}\Gamma_{l}I_{f}^{T}\bar{I},N_{1}≜\left[\begin{array}{cc} {\overset{˘}{N}}_{1} & 0 \end{array}\right],\\ \; & {\tilde{\Psi }}_{B1}≜\left[\begin{array}{ccc} \bar{M}\bar{W}\bar{A}+\left(1-\bar{\beta }\right)\left({\overset{˘}{K}}_{P}+{\overset{˘}{K}}_{D}\right) & -\left(1-\bar{\beta }\right)\left({\overset{˘}{K}}_{P}+{\overset{˘}{K}}_{D}\right) & \left(1-\bar{\beta }\right){\hat{B}}_{D1}\\ \bar{\beta }\overset{˘}{L}\bar{C} & P_{2}\bar{A}-\overset{˘}{L}\bar{C} & 0\\ 0 & 0 & 0\\ 0 & 0 & 0\\ -\tilde{\beta }\left({\overset{˘}{K}}_{P}+{\overset{˘}{K}}_{D}\right) & \tilde{\beta }\left({\overset{˘}{K}}_{P}+{\overset{˘}{K}}_{D}\right) & -\tilde{\beta }{\hat{B}}_{D1}\\ 0 & 0 & 0\\ 0 & 0 & 0\\ \tilde{\beta }\overset{˘}{L}\bar{C} & 0 & 0 \end{array}\right],\\ \; & P_{\bar{M}}≜\mathrm{diag}\{-\bar{M}\bar{W}-{\bar{W}}^{T}{\bar{M}}^{T}+P_{1},-P_{2},-\bar{M}\bar{W}-{\bar{W}}^{T}{\bar{M}}^{T}+P_{1},-P_{2},-\bar{M}\bar{W}\\ \; & \;\;\;\;\;-{\bar{W}}^{T}{\bar{M}}^{T}+P_{1},-P_{2},-\bar{M}\bar{W}-{\bar{W}}^{T}{\bar{M}}^{T}+P_{1},-P_{2}\},\tilde{M}≜\mathrm{diag}\left\{\bar{M}\bar{W},P_{2}\right\},\\ \; & {\overset{˘}{E}}_{1}≜\mathrm{diag}\left\{E_{1},E_{1}\right\},E_{1}≜{\left[E^{T}\;\underset{s}{\underset{︸}{\begin{array}{cccc} 0 & 0 & \cdots & 0 \end{array}}}\right]}^{T},N_{1}≜\left[N\;\underset{s}{\underset{︸}{\begin{array}{cccc} 0 & 0 & \cdots & 0 \end{array}}}\right],\\ \; & {\hat{B}}_{D1}≜\left[\left[\begin{array}{cc} {\overset{˘}{K}}_{I}-{\overset{˘}{K}}_{D} & -{\overset{˘}{K}}_{I}+{\overset{˘}{K}}_{D} \end{array}\right]\;\;\underset{N-1}{\underset{︸}{\left[\begin{array}{cc} {\overset{˘}{K}}_{I} & -{\overset{˘}{K}}_{I} \end{array}\right]\;\;\cdots \;\;\left[\begin{array}{cc} {\overset{˘}{K}}_{I} & -{\overset{˘}{K}}_{I} \end{array}\right]}}\right],\\ \; & {\overset{˘}{\Upsilon }}_{M21}≜\mathrm{diag}\left\{\sqrt{w_{0}}P{\bar{G}}_{1},\sqrt{{℧}_{1}v_{0}}{\hat{L}}_{1}D,\sqrt{{℧}_{2}\left(\theta^{2}/4\right)}{\hat{L}}_{1},\sqrt{{℧}_{4}\left(\theta^{2}/4\right)}P{\bar{B}}_{1}\right\},\\ \; & {\overset{˘}{\Upsilon }}_{M31}≜\mathrm{diag}\left\{0,0,\sqrt{{℧}_{3}\left(1/{\left(1-2\bar{϶}\right)}^{2}\right){ℑ}^{2}\pi }{\hat{L}}_{1},\sqrt{{℧}_{5}\left(1/{\left(1-2\bar{϶}\right)}^{2}\right){ℑ}^{2}\pi }P{\bar{B}}_{1}\right\}. \end{array}$$

*Furthermore, the upper bound of the ultimate upper bound of $E\{∥z\left(k\right){∥}^{2}\}$ is minimized by solving the following optimization issue:*

(57) $$\begin{array}{c} \min_{\mathrm{subject}\mathrm{to}\;(53)–(56)}\mathrm{tr}\left(\overset{˘}{\Upsilon }\right). \end{array}$$

*Gain expressions are denoted as*

(58) $$\begin{array}{c} K_{P}={\bar{M}}_{1}^{-1}{\bar{K}}_{P},K_{I}={\bar{M}}_{1}^{-1}{\bar{K}}_{I},K_{D}={\bar{M}}_{1}^{-1}{\bar{K}}_{D},L=P_{2}^{-1}\overset{˘}{L}. \end{array}$$

**Proof of Theorem** **2.** By using Schur Complement Lemma, we see that the following inequality holds as long as (53) holds:

(59) $$\begin{array}{c} \iota_{l}^{T}{\bar{I}}_{f1}^{T}P{\bar{I}}_{f1}\iota_{l}<\nu . \end{array}$$

We notice that (34) holds as long as the inequality below holds:

(60) $$\begin{array}{c} \bar{\Pi }≜\left[\begin{array}{cc} {\bar{\Pi }}_{11} & \Pi_{12}\\ {}* & \Pi_{22} \end{array}\right]<0 \end{array}$$

where

$$\begin{array}{cc} {\bar{\Pi }}_{11}≜ & -P+{\left(\overset{˘}{A}+\bar{\alpha }\Delta {\overset{˘}{A}}_{1}\right)}^{T}P\left(\overset{˘}{A}+\bar{\alpha }\Delta {\overset{˘}{A}}_{1}\right)+{\tilde{\alpha }}^{2}\Delta {\overset{˘}{A}}_{1}^{T}P\Delta {\overset{˘}{A}}_{1}+{\tilde{\beta }}^{2}{\bar{L}}_{C}^{T}P{\bar{L}}_{C}+{\tilde{\beta }}^{2}{\overset{˘}{B}}_{L}^{T}P{\overset{˘}{B}}_{L}\\ \; & +\sum_{l=1}^{\xi }\nu {\bar{I}}^{T}I_{f}\Gamma_{l}I_{f}^{T}\bar{I}+\sum_{j=1}^{N}Q_{j}. \end{array}$$

(60) is equivalent to the inequality below:

(61) $$\begin{array}{c} \bar{\Pi }=\Psi_{0}+\Psi_{1}^{T}P\Psi_{1}+\Psi_{2}^{T}P\Psi_{2}+\Psi_{3}^{T}P\Psi_{3}+\Psi_{4}^{T}P\Psi_{4}<0 \end{array}$$

where

$$\begin{array}{cc} \Psi_{1}≜ & \left[\begin{array}{cc} \overset{˘}{A}+\bar{\alpha }\Delta {\overset{˘}{A}}_{1}\; & \left(1-\bar{\beta }\right){\overset{˘}{B}}_{D} \end{array}\right],\Psi_{2}≜\left[\begin{array}{cc} \tilde{\alpha }\Delta {\overset{˘}{A}}_{1} & 0 \end{array}\right],\Psi_{3}≜\left[\begin{array}{cc} \tilde{\beta }{\overset{˘}{B}}_{L} & -\tilde{\beta }{\overset{˘}{B}}_{D} \end{array}\right],\Psi_{4}≜\left[\begin{array}{cc} \tilde{\beta }{\bar{L}}_{C} & 0 \end{array}\right]. \end{array}$$

That is,

(62) $$\begin{array}{c} \bar{\Pi }=\Psi_{0}+\left[\begin{array}{cccc} \Psi_{1}^{T} & \Psi_{2}^{T} & \Psi_{3}^{T} & \Psi_{4}^{T} \end{array}\right]\left(I_{\left(4\right)}⊗P\right)\left[\begin{array}{c} \Psi_{1}\\ \Psi_{2}\\ \Psi_{3}\\ \Psi_{4} \end{array}\right]<0. \end{array}$$

Adopting Schur Complement Lemma, (62) holds as long as the inequality below holds:

(63) $$\begin{array}{c} \left[\begin{array}{ccccc} \Psi_{0} & * & * & * & *\\ \Psi_{1} & -P^{-1} & * & * & *\\ \Psi_{2} & 0 & -P^{-1} & * & *\\ \Psi_{3} & 0 & 0 & -P^{-1} & *\\ \Psi_{4} & 0 & 0 & 0 & -P^{-1} \end{array}\right]<0, \end{array}$$

i.e.,

(64) $$\begin{array}{c} \left[\begin{array}{cccccc} \Psi_{01} & * & * & * & * & *\\ 0 & -Q & * & * & * & *\\ \overset{˘}{A}+\bar{\alpha }\Delta {\overset{˘}{A}}_{1}\; & \left(1-\bar{\beta }\right){\overset{˘}{B}}_{D} & -P^{-1} & * & * & *\\ \tilde{\alpha }\Delta {\overset{˘}{A}}_{1} & 0 & 0 & -P^{-1} & * & *\\ \tilde{\beta }{\overset{˘}{B}}_{L} & -\tilde{\beta }{\overset{˘}{B}}_{D} & 0 & 0 & -P^{-1} & *\\ \tilde{\beta }{\bar{L}}_{C} & 0 & 0 & 0 & 0 & -P^{-1} \end{array}\right]<0. \end{array}$$

Divide (64) into a sum form with a certain term and an uncertain term:

(65) $$\begin{array}{c} \Psi_{a}+\left[\begin{array}{cc} 0 & 0\\ \Delta A & 0 \end{array}\right]+{\left[\begin{array}{cc} 0 & 0\\ \Delta A & 0 \end{array}\right]}^{T}<0 \end{array}$$

where

$$\begin{array}{c} \Psi_{a}≜\left[\begin{array}{cccccc} \Psi_{01} & * & * & * & * & *\\ 0 & -Q & * & * & * & *\\ \overset{˘}{A}\; & \left(1-\bar{\beta }\right){\overset{˘}{B}}_{D} & -P^{-1} & * & * & *\\ 0 & 0 & 0 & -P^{-1} & * & *\\ \tilde{\beta }{\overset{˘}{B}}_{L} & -\tilde{\beta }{\overset{˘}{B}}_{D} & 0 & 0 & -P^{-1} & *\\ \tilde{\beta }{\bar{L}}_{C} & 0 & 0 & 0 & 0 & -P^{-1} \end{array}\right],\Delta A≜\left[\begin{array}{cc} \bar{\alpha }\Delta {\overset{˘}{A}}_{1} & 0\\ \tilde{\alpha }\Delta {\overset{˘}{A}}_{1} & 0\\ 0 & 0\\ 0 & 0 \end{array}\right]. \end{array}$$

Noting that $\Delta {\overset{˘}{A}}_{1}={\overset{˘}{E}}_{1}\overset{˘}{F}{\overset{˘}{N}}_{1}$, we acquire

$$\begin{array}{c} \Delta A=E_{1}\overset{˘}{F}N_{1} \end{array}$$

where $\overset{˘}{F}≜\mathrm{diag}\left\{F,F\right\}$.

(65) is transformed into the inequality below:

(66) $$\begin{array}{c} \Psi_{a}+{\tilde{E}}_{1}\overset{˘}{F}{\tilde{N}}_{1}+{\tilde{N}}_{1}^{T}{\overset{˘}{F}}^{T}{\tilde{E}}_{1}^{T}<0 \end{array}$$

where

$$\begin{array}{c} {\tilde{E}}_{1}≜\left[\begin{array}{c} 0\\ E_{1} \end{array}\right],{\tilde{N}}_{1}≜\left[\begin{array}{cc} N_{1} & 0 \end{array}\right]. \end{array}$$

By utilizing S-procedure Lemma, we know that (66) holds as long as the inequality below holds:

(67) $$\begin{array}{cc} \; & \Psi_{a}+\zeta^{-1}{\tilde{E}}_{1}{\tilde{E}}_{1}^{T}+\zeta {\tilde{N}}_{1}^{T}{\tilde{N}}_{1}<0. \end{array}$$

Adopting Schur Complement Lemma, (67) is satisfies as long as the inequality below holds:

(68) $$\begin{array}{cc} \; & \left[\begin{array}{ccc} \Psi_{a} & * & *\\ {\tilde{E}}_{1}^{T} & -\zeta I & *\\ \zeta {\tilde{N}}_{1} & 0 & -\zeta I \end{array}\right]<0, \end{array}$$

i.e.,

(69) $$\begin{array}{cc} \; & \left[\begin{array}{cccc} \Psi_{0} & * & * & *\\ \Psi_{B} & -I_{\left(4\right)}⊗P^{-1} & * & *\\ 0 & E_{1}^{T} & -\zeta I & *\\ \zeta N_{1} & 0 & 0 & -\zeta I \end{array}\right]<0 \end{array}$$

where

$$\begin{array}{c} \Psi_{B}≜\left[\begin{array}{cc} \overset{˘}{A} & \left(1-\bar{\beta }\right){\overset{˘}{B}}_{D}\\ 0 & 0\\ \tilde{\beta }{\overset{˘}{B}}_{L} & -\tilde{\beta }{\overset{˘}{B}}_{D}\\ \tilde{\beta }{\bar{L}}_{C} & 0 \end{array}\right]. \end{array}$$

Notice that the expansion form of (69) is shown in the following inequality:

(70) $$\begin{array}{cc} \; & \left[\begin{array}{cccccccc} \Psi_{01} & * & * & * & * & * & * & *\\ 0 & -Q & * & * & * & * & * & *\\ \overset{˘}{A} & \left(1-\bar{\beta }\right){\overset{˘}{B}}_{D} & -P^{-1} & * & * & * & * & *\\ 0 & 0 & 0 & -P^{-1} & * & * & * & *\\ \tilde{\beta }{\overset{˘}{B}}_{L} & -\tilde{\beta }{\overset{˘}{B}}_{D} & 0 & 0 & -P^{-1} & * & * & *\\ \tilde{\beta }{\bar{L}}_{C} & 0 & 0 & 0 & 0 & -P^{-1} & * & *\\ 0 & 0 & \bar{\alpha }{\overset{˘}{E}}_{1}^{T} & \tilde{\alpha }{\overset{˘}{E}}_{1}^{T} & 0 & 0 & -\zeta I & *\\ \zeta {\overset{˘}{N}}_{1} & 0 & 0 & 0 & 0 & 0 & 0 & -\zeta I \end{array}\right]<0. \end{array}$$

Left- and right-multiplying (69) by $\mathrm{diag}\{I,I_{\left(4\right)}⊗\tilde{M},I,I\}$ and $\mathrm{diag}\{I,I_{\left(4\right)}⊗{\tilde{M}}^{T},I,I\}$, we derive

(71) $$\begin{array}{cc} \; & \left[\begin{array}{cccc} \Psi_{0} & * & * & *\\ \left(I_{\left(4\right)}⊗\tilde{M}\right)\Psi_{B} & -I_{\left(4\right)}⊗\left(\tilde{M}P^{-1}{\tilde{M}}^{T}\right) & * & *\\ 0 & E_{1}^{T}\left(I_{\left(4\right)}⊗{\tilde{M}}^{T}\right) & -\zeta I & *\\ \zeta N_{1} & 0 & 0 & -\zeta I \end{array}\right]<0. \end{array}$$

(71) is the same as the detailed form below:

(72) $$\begin{array}{cc} \; & \left[\begin{array}{cccccccc} \Psi_{01} & * & * & * & * & * & * & *\\ 0 & -Q & * & * & * & * & * & *\\ \tilde{M}\overset{˘}{A} & \left(1-\bar{\beta }\right)\tilde{M}{\overset{˘}{B}}_{D} & -\tilde{M}P^{-1}{\tilde{M}}^{T} & * & * & * & * & *\\ 0 & 0 & 0 & -\tilde{M}P^{-1}{\tilde{M}}^{T} & * & * & * & *\\ \tilde{\beta }\tilde{M}{\overset{˘}{B}}_{L} & -\tilde{\beta }\tilde{M}{\overset{˘}{B}}_{D} & 0 & 0 & -\tilde{M}P^{-1}{\tilde{M}}^{T} & * & * & *\\ \tilde{\beta }\tilde{M}{\bar{L}}_{C} & 0 & 0 & 0 & 0 & -\tilde{M}P^{-1}{\tilde{M}}^{T} & * & *\\ 0 & 0 & \bar{\alpha }{\overset{˘}{E}}_{1}^{T}{\tilde{M}}^{T} & \tilde{\alpha }{\overset{˘}{E}}_{1}^{T}{\tilde{M}}^{T} & 0 & 0 & -\zeta I & *\\ \zeta {\overset{˘}{N}}_{1} & 0 & 0 & 0 & 0 & 0 & 0 & -\zeta I \end{array}\right]\\ \; & <0. \end{array}$$

Noticing that

$$\begin{array}{cc} \; & \bar{M}\bar{W}+{\bar{W}}^{T}{\bar{M}}^{T}-\bar{M}\bar{W}P_{1}^{-1}{\bar{W}}^{T}{\bar{M}}^{T}-P_{1}=-(\bar{M}\bar{W}-P_{1})P_{1}^{-1}(\bar{M}\bar{W}-P_{1}{)}^{T}\leq 0, \end{array}$$

we know that

(73) $$\begin{array}{cc} \; & -\bar{M}\bar{W}P_{1}^{-1}{\bar{W}}^{T}{\bar{M}}^{T}\leq -\bar{M}\bar{W}-{\bar{W}}^{T}{\bar{M}}^{T}+P_{1}. \end{array}$$

With regard to (73), one yields

(74) $$\begin{array}{cc} \; & -I_{\left(4\right)}⊗\left(\tilde{M}P^{-1}{\tilde{M}}^{T}\right)\\ = & \mathrm{diag}\{-\bar{M}\bar{W}P_{1}^{-1}{\bar{W}}^{T}{\bar{M}}^{T},-P_{2},-\bar{M}\bar{W}P_{1}^{-1}{\bar{W}}^{T}{\bar{M}}^{T},-P_{2},-\bar{M}\bar{W}P_{1}^{-1}{\bar{W}}^{T}{\bar{M}}^{T},-P_{2},\\ \; & \;\;-\bar{M}\bar{W}P_{1}^{-1}{\bar{W}}^{T}{\bar{M}}^{T},-P_{2}\}\\ \leq & \mathrm{diag}\{-\bar{M}\bar{W}-{\bar{W}}^{T}{\bar{M}}^{T}+P_{1},-P_{2},-\bar{M}\bar{W}-{\bar{W}}^{T}{\bar{M}}^{T}+P_{1},-P_{2},-\bar{M}\bar{W}\\ \; & \;\;-{\bar{W}}^{T}{\bar{M}}^{T}+P_{1},-P_{2},-\bar{M}\bar{W}-{\bar{W}}^{T}{\bar{M}}^{T}+P_{1},-P_{2}\}=P_{\bar{M}}. \end{array}$$

In consideration of (74), one recognizes that (71) is met if the inequality below holds:

(75) $$\begin{array}{cc} \; & \left[\begin{array}{cccc} \Psi_{0} & * & * & *\\ \left(I_{\left(4\right)}⊗\tilde{M}\right)\Psi_{B} & P_{\bar{M}} & * & *\\ 0 & E_{1}^{T}\left(I_{\left(4\right)}⊗{\tilde{M}}^{T}\right) & -\zeta I & *\\ \zeta N_{1} & 0 & 0 & -\zeta I \end{array}\right]<0. \end{array}$$

For a term in (75), we obtain

(76) $$\begin{array}{cc} \; & \left(I_{\left(4\right)}⊗\tilde{M}\right)\Psi_{B}\\ = & \left[\begin{array}{cc} \tilde{M}\overset{˘}{A} & \left(1-\bar{\beta }\right)\tilde{M}{\overset{˘}{B}}_{D}\\ 0 & 0\\ \tilde{\beta }\tilde{M}{\overset{˘}{B}}_{L} & -\tilde{\beta }\tilde{M}{\overset{˘}{B}}_{D}\\ \tilde{\beta }\tilde{M}{\bar{L}}_{C} & 0 \end{array}\right]\\ = & \left[\begin{array}{ccc} \bar{M}\bar{W}\bar{A}+\left(1-\bar{\beta }\right)\bar{M}\bar{W}\bar{B}\left(K_{P}+K_{D}\right) & -\left(1-\bar{\beta }\right)\bar{M}\bar{W}\bar{B}\left(K_{P}+K_{D}\right) & \left(1-\bar{\beta }\right){\overset{´}{B}}_{D}\\ \bar{\beta }P_{2}L\bar{C} & P_{2}\bar{A}-P_{2}L\bar{C} & 0\\ 0 & 0 & 0\\ 0 & 0 & 0\\ -\tilde{\beta }\bar{M}\bar{W}\bar{B}\left(K_{P}+K_{D}\right) & \tilde{\beta }\bar{M}\bar{W}\bar{B}\left(K_{P}+K_{D}\right) & -\tilde{\beta }{\overset{´}{B}}_{D}\\ 0 & 0 & 0\\ 0 & 0 & 0\\ \tilde{\beta }P_{2}L\bar{C} & 0 & 0 \end{array}\right] \end{array}$$

where

$$\begin{array}{c} {\overset{´}{B}}_{D}≜\left[\bar{M}\bar{W}\bar{B}\left[\begin{array}{cc} K_{I}-K_{D} & -K_{I}+K_{D} \end{array}\right]\;\;\underset{N-1}{\underset{︸}{\bar{M}\bar{W}\bar{B}\left[\begin{array}{cc} K_{I} & -K_{I} \end{array}\right]\;\;\cdots \;\;\bar{M}\bar{W}\bar{B}\left[\begin{array}{cc} K_{I} & -K_{I} \end{array}\right]}}\right]. \end{array}$$

Using the properties including (1) inverse of matrix multiplication (${\left(ZX\right)}^{-1}=X^{-1}Z^{-1}$ where *Z* and *X* are square matrices with proper sizes), (2) permissible order exchange of inverse and transpose (${\left(Z^{T}\right)}^{-1}={\left(Z^{-1}\right)}^{T}$), and (3) orthogonality ($YY^{⊥}=0$ where *Y* is a matrix with appropriate dimension, similarly, $Y^{T}{\left(Y^{T}\right)}^{⊥}=0$, that is, ${\left({\left(Y^{T}\right)}^{⊥}\right)}^{T}Y=0$), we have

(77) $$\begin{array}{cc} \bar{W}\bar{B}= & {\left[\begin{array}{cc} \bar{B}({\bar{B}}^{T}\bar{B}{)}^{-1} & {\left({\bar{B}}^{T}\right)}^{⊥} \end{array}\right]}^{T}\bar{B}=\left[\begin{array}{c} {\left(\bar{B}({\bar{B}}^{T}\bar{B}{)}^{-1}\right)}^{T}\\ {\left({\left({\bar{B}}^{T}\right)}^{⊥}\right)}^{T} \end{array}\right]\bar{B}\\ = & \left[\begin{array}{c} {\left(\bar{B}{\bar{B}}^{-1}{\left({\bar{B}}^{T}\right)}^{-1}\right)}^{T}\\ {\left({\left({\bar{B}}^{T}\right)}^{⊥}\right)}^{T} \end{array}\right]\bar{B}=\left[\begin{array}{c} {\left({\left({\bar{B}}^{-1}\right)}^{T}\right)}^{T}\\ {\left({\left({\bar{B}}^{T}\right)}^{⊥}\right)}^{T} \end{array}\right]\bar{B}=\left[\begin{array}{c} {\bar{B}}^{-1}\\ {\left({\left({\bar{B}}^{T}\right)}^{⊥}\right)}^{T} \end{array}\right]\bar{B}=\left[\begin{array}{c} I\\ 0 \end{array}\right]. \end{array}$$

Considering (77), we yield the expressions as follows:

$$\begin{array}{cc} \; & \bar{M}\bar{W}\bar{B}K_{P}=\bar{M}\left[\begin{array}{c} I\\ 0 \end{array}\right]K_{P}=\left[\begin{array}{c} {\bar{M}}_{1}K_{P}\\ 0 \end{array}\right],\bar{M}\bar{W}\bar{B}K_{I}=\bar{M}\left[\begin{array}{c} I\\ 0 \end{array}\right]K_{I}=\left[\begin{array}{c} {\bar{M}}_{1}K_{I}\\ 0 \end{array}\right],\\ \; & \bar{M}\bar{W}\bar{B}K_{D}=\bar{M}\left[\begin{array}{c} I\\ 0 \end{array}\right]K_{D}=\left[\begin{array}{c} {\bar{M}}_{1}K_{D}\\ 0 \end{array}\right]. \end{array}$$

In order to eliminate nonlinear terms, a variable transformation is conducted below:

(78) $$\begin{array}{c} {\bar{K}}_{P}≜{\bar{M}}_{1}K_{P},{\bar{K}}_{I}≜{\bar{M}}_{1}K_{I},{\bar{K}}_{D}≜{\bar{M}}_{1}K_{D},\overset{˘}{L}≜P_{2}L. \end{array}$$

Then, the following expressions are derived:

(79) $$\begin{array}{cc} \; & \bar{M}\bar{W}\bar{B}K_{P}=\left[\begin{array}{c} {\bar{K}}_{P}\\ 0 \end{array}\right]={\overset{˘}{K}}_{P},\bar{M}\bar{W}\bar{B}K_{I}=\left[\begin{array}{c} {\bar{K}}_{I}\\ 0 \end{array}\right]={\overset{˘}{K}}_{I},\bar{M}\bar{W}\bar{B}K_{D}=\left[\begin{array}{c} {\bar{K}}_{D}\\ 0 \end{array}\right]={\overset{˘}{K}}_{D}. \end{array}$$

Combining (76) and (79) with (75), we notice that (75) is the same as (54).

According to (31), (52) and (55), we have the following inequality:

(80) $$\begin{array}{cc} E\{∥z\left(k\right){∥}^{2}\}= & E\left\{{\bar{\eta }}^{T}\left(k\right){\tilde{H}}^{T}\tilde{H}\bar{\eta }\left(k\right)\right\}<E\left\{{\bar{\eta }}^{T}\left(k\right)P\bar{\eta }\left(k\right)\right\}<E\{\lambda_{\max}\left(P\right)∥\bar{\eta }\left(k\right){∥}^{2}\}\\ \leq & c_{0}\lambda_{\max}\left(P\right)(\frac{1}{h_{0}}{)}^{k+1}\underset{i\leq 0}{\sup}E\{∥\bar{\eta }\left(i\right){∥}^{2}\}+\frac{h_{0}\lambda_{\max}\left(P\right)}{\chi (h_{0}-1)}\mathrm{tr}\left(\Upsilon \right). \end{array}$$

Then we know that $E\{∥z\left(k\right){∥}^{2}\}$ reaches EUBMS performance, and its ultimate upper bound is $\frac{h_{0}\lambda_{\max}\left(P\right)}{\chi (h_{0}-1)}\mathrm{tr}\left(\Upsilon \right)$.

We note easily that

(81) $$\begin{array}{cc} \Upsilon = & \Upsilon_{1}^{T}\left(I_{\left(4\right)}⊗P\right)\Upsilon_{1}+\Upsilon_{2}^{T}\left(I_{\left(4\right)}⊗P\right)\Upsilon_{2}\\ = & \Upsilon_{1}^{T}\left(I_{\left(4\right)}⊗P\right){\left(I_{\left(4\right)}⊗P\right)}^{-1}\left(I_{\left(4\right)}⊗P\right)\Upsilon_{1}+\Upsilon_{2}^{T}\left(I_{\left(4\right)}⊗P\right){\left(I_{\left(4\right)}⊗P\right)}^{-1}\left(I_{\left(4\right)}⊗P\right)\Upsilon_{2}\\ = & \Upsilon_{P1}^{T}{\left(I_{\left(4\right)}⊗P\right)}^{-1}\Upsilon_{P1}+\Upsilon_{P2}^{T}{\left(I_{\left(4\right)}⊗P\right)}^{-1}\Upsilon_{P2} \end{array}$$

where

$$\begin{array}{cc} \Upsilon_{1}≜ & \mathrm{diag}\{\sqrt{w_{0}}{\bar{G}}_{1},\sqrt{{℧}_{1}v_{0}}{\bar{L}}_{D},\sqrt{{℧}_{2}\left(\theta^{2}/4\right)}{\bar{L}}_{1},\sqrt{{℧}_{4}\left(\theta^{2}/4\right)}{\bar{B}}_{1}\},\\ \Upsilon_{2}≜ & \mathrm{diag}\{0,0,\sqrt{{℧}_{3}\left(1/{\left(1-2\bar{϶}\right)}^{2}\right){ℑ}^{2}\pi }{\bar{L}}_{1},\sqrt{{℧}_{5}\left(1/{\left(1-2\bar{϶}\right)}^{2}\right){ℑ}^{2}\pi }{\bar{B}}_{1}\},\\ \Upsilon_{P1}≜ & \mathrm{diag}\{\sqrt{w_{0}}P{\bar{G}}_{1},\sqrt{{℧}_{1}v_{0}}P{\bar{L}}_{D},\sqrt{{℧}_{2}\left(\theta^{2}/4\right)}P{\bar{L}}_{1},\sqrt{{℧}_{4}\left(\theta^{2}/4\right)}P{\bar{B}}_{1}\},\\ \Upsilon_{P2}≜ & \mathrm{diag}\{0,0,\sqrt{{℧}_{3}\left(1/{\left(1-2\bar{϶}\right)}^{2}\right){ℑ}^{2}\pi }P{\bar{L}}_{1},\sqrt{{℧}_{5}\left(1/{\left(1-2\bar{϶}\right)}^{2}\right){ℑ}^{2}\pi }P{\bar{B}}_{1}\}. \end{array}$$

Regarding $\overset{˘}{L}=P_{2}L$ in (78) and ${\hat{L}}_{1}={\left[\begin{array}{cc} 0 & {\overset{˘}{L}}^{T} \end{array}\right]}^{T}$ in Theorem 2, we have

$$\begin{array}{cc} \; & P{\bar{L}}_{D}=\left[\begin{array}{cc} P_{1} & 0\\ 0 & P_{2} \end{array}\right]\left[\begin{array}{c} 0\\ LD \end{array}\right]=\left[\begin{array}{c} 0\\ P_{2}L \end{array}\right]D={\hat{L}}_{1}D,P{\bar{L}}_{1}=\left[\begin{array}{cc} P_{1} & 0\\ 0 & P_{2} \end{array}\right]\left[\begin{array}{c} 0\\ L \end{array}\right]=\left[\begin{array}{c} 0\\ P_{2}L \end{array}\right]={\hat{L}}_{1}, \end{array}$$

and then,

$$\begin{array}{c} \Upsilon_{P1}={\overset{˘}{\Upsilon }}_{M21},\Upsilon_{P2}={\overset{˘}{\Upsilon }}_{M31}. \end{array}$$

According to (81), we have

(82) $$\begin{array}{cc} \Upsilon = & {\overset{˘}{\Upsilon }}_{M21}^{T}{\left(I_{\left(4\right)}⊗P\right)}^{-1}{\overset{˘}{\Upsilon }}_{M21}+{\overset{˘}{\Upsilon }}_{M31}^{T}{\left(I_{\left(4\right)}⊗P\right)}^{-1}{\overset{˘}{\Upsilon }}_{M31}. \end{array}$$

Taking (56) and (82) into account and using Schur Complement Lemma, we get that

(83) $$\begin{array}{c} \Upsilon <\overset{˘}{\Upsilon }. \end{array}$$

With (83), the bound of the ultimate upper bound of $E\{∥z\left(k\right){∥}^{2}\}$ is $\frac{h_{0}\lambda_{\max}\left(P\right)}{\chi (h_{0}-1)}\mathrm{tr}\left(\overset{˘}{\Upsilon }\right)$, which is minimized by solving the optimization issue (57). The proof is finished now. □

**Remark** **1.** *Until now, an observer-based PID controller (25) has been designed for uncertain nonlinear systems with integral measurements and DoS attacks using a BES. In Theorem 1, the performance has been analyzed of the closed-loop system (i.e., EUBMS performance). In Theorem 2, a sufficient condition has been presented for designing the desired controller which makes both the closed-loop system (31) and the controlled output realize EUBMS performance, and ensures that the ultimate upper bound of the controlled output is bounded and such a bound is minimized. Note that all the critical parameters have been involved in Theorem 2 such as system parameters, parameters in variance of stochastic nonlinearities, deterministic matrices in uncertain parameter, occurring probabilities of parameter uncertainties, DoS attacks and bit flippings, length of BBS, range of the transmitted signal, and variances of process noise and measurement noise.*

**Remark** **2.** *In this section, the main work of this paper is accomplished with designing a desired observer-based PID controller. Compared with existing control methods, novelties of the obtained results in this paper are (1) a new and comprehensive framework of observer-based PID control issue is established which contain integral measurements, usage of BES in both the sensor-to-observer channel and the controller-to-actuator channel, randomly occurring uncertainties and DoS attacks, stochastic nonlinearities, and bounded stochastic noises; (2) a new performance analysis process is executed in this paper which assures that both the closed-loop system and the controlled output possess EUBMS performance; and (3) a new observer-based PID control approach is designed with a minimized upper bound of the ultimate upper bound of the controlled output, and the gain matrices of such a controller are attained conveniently via settling an optimized issue with matrix inequalities as constraints.*

## 4. Simulation Examples

In this section, two simulation examples are conducted to testify the availability of the developed observer-based PID control approach.

**Example** **1.** *For uncertain nonlinear system (1), the parameters are set as follows.*

$$\begin{array}{cc} \; & n_{x}=2,n_{u}=1,n_{z}=2,n_{w}=1,n_{y}=1,n_{v}=1,s=2,w_{0}=0.2,v_{0}=0.3,\\ \; & A=\left[\begin{array}{cc} 0.26 & 0.09\\ 0.18 & -0.32 \end{array}\right],B=\left[\begin{array}{c} -1.8\\ -1.3 \end{array}\right],G=\left[\begin{array}{c} 0.1\\ -0.2 \end{array}\right],H=\left[\begin{array}{cc} -0.4 & 0.1\\ 0.1 & 0.1 \end{array}\right],N=\left[\begin{array}{cc} 1 & 1\\ 1 & 1 \end{array}\right],\\ \; & C=\left[\begin{array}{cc} 1.2 & 2.8 \end{array}\right],D=0.2,E=0.01I_{\left(2\right)},\bar{\alpha }=0.8,\bar{϶}=0.01,\bar{\beta }=0.15,N=3,\\ \; & \theta =0.048,ð=6,{℘}_{1}={℘}_{2}={℘}_{3}={℘}_{4}={℘}_{5}={℘}_{6}={℘}_{7}={℘}_{8}=0.01. \end{array}$$

*The stochastic nonlinear function is selected as*

$$\begin{array}{c} f\left(k,x\left(k\right)\right)=\left[\begin{array}{c} 0.04\\ 0.05 \end{array}\right]\times \left(0.2x_{1}\left(k\right)\phi_{1}\left(k\right)+0.3x_{2}\left(k\right)\phi_{2}\left(k\right)\right) \end{array}$$

*where $x_{i}\left(k\right)$ (i=1,2) denotes the ith component of x(k), and $\phi_{i}\left(k\right)$ (i=1,2) denote uncorrelated Gaussian white noise processes with zero mean and unity variance. Note that $\phi_{i}\left(k\right)$ (i=1,2) are also uncorrelated with w(k) and v(k). We see that such a kind of stochastic nonlinearity fulfills*

$$\begin{array}{cc} \; & E\{f(k,x(k))|x(k)\}=0,\\ \; & E\left\{f\left(k,x\left(k\right)\right)f^{T}\left(k,x\left(k\right)\right)|x\left(k\right)\right\}=\left[\begin{array}{c} 0.04\\ 0.05 \end{array}\right]{\left[\begin{array}{c} 0.04\\ 0.05 \end{array}\right]}^{T}E\left\{x^{T}\left(k\right)\left[\begin{array}{cc} 0.04 & 0\\ 0 & 0.09 \end{array}\right]x\left(k\right)\right\}. \end{array}$$

*By resolving the optimization issue (57) constrained by matrix inequalities (53)–(56), the gains of the observer-based PID controller (25) are acquired below.*

$$\begin{array}{cc} \; & L=10^{-3}\times {\left[\begin{array}{cccccc} -0.0045 & -0.0113 & 0.1939 & 0.1939 & 0.1883 & -0.0005 \end{array}\right]}^{T},\\ \; & K_{P}=\left[\begin{array}{cccccc} -6.2254 & -6.2254 & -6.2254 & -0.8567 & -6.2254 & -6.2254 \end{array}\right],\\ \; & K_{I}=10^{-3}\times \left[\begin{array}{cccccc} -0.0085 & 0.0006 & -0.1339 & -0.1844 & -0.0142 & -0.1829 \end{array}\right],\\ \; & K_{D}=10^{-3}\times \left[\begin{array}{cccccc} -0.0163 & -0.0004 & -0.4637 & -0.1429 & -0.0317 & -0.4832 \end{array}\right]. \end{array}$$

**Figure 2:**
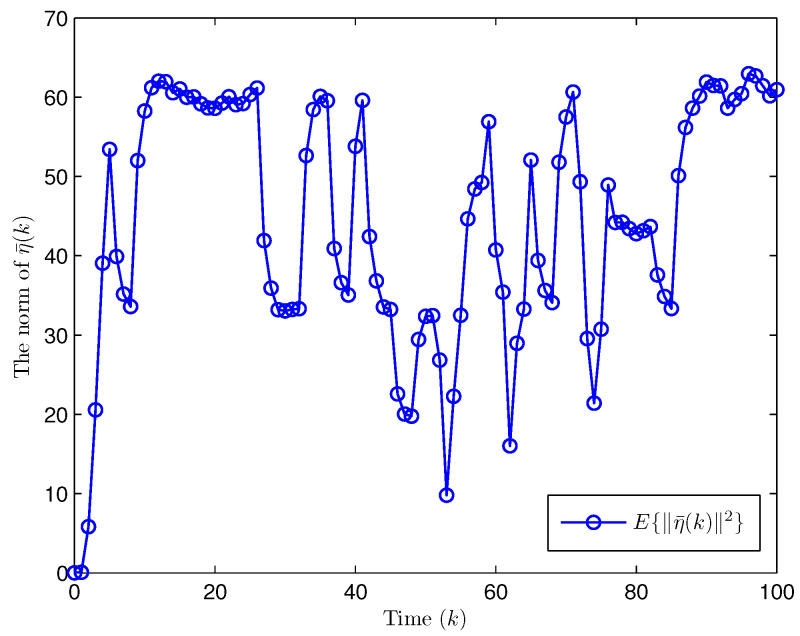
Evolution of the expectation of the norm of $\bar{\eta }\left(k\right)$ (Example 1).

**Figure 3:**
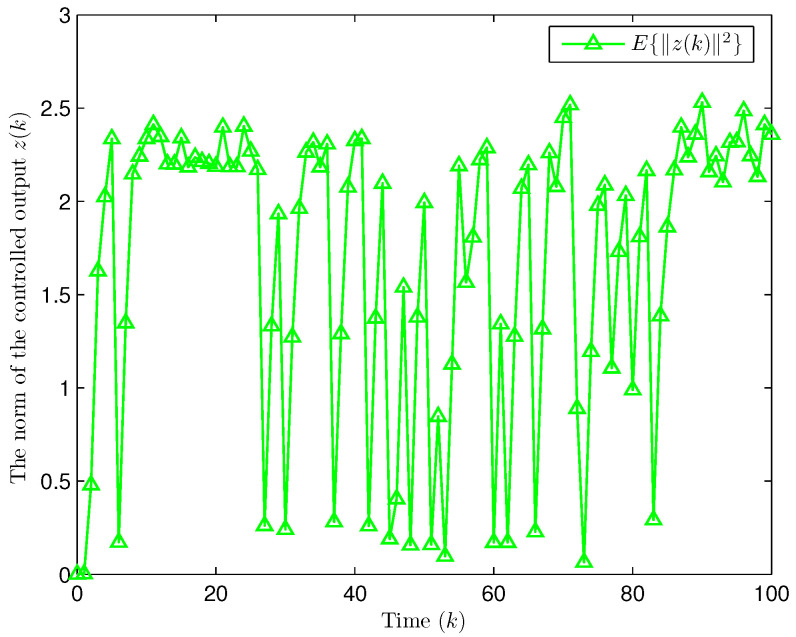
Evolution of the expectation of the norm of z(k) (Example 1).

**Figure 4:**
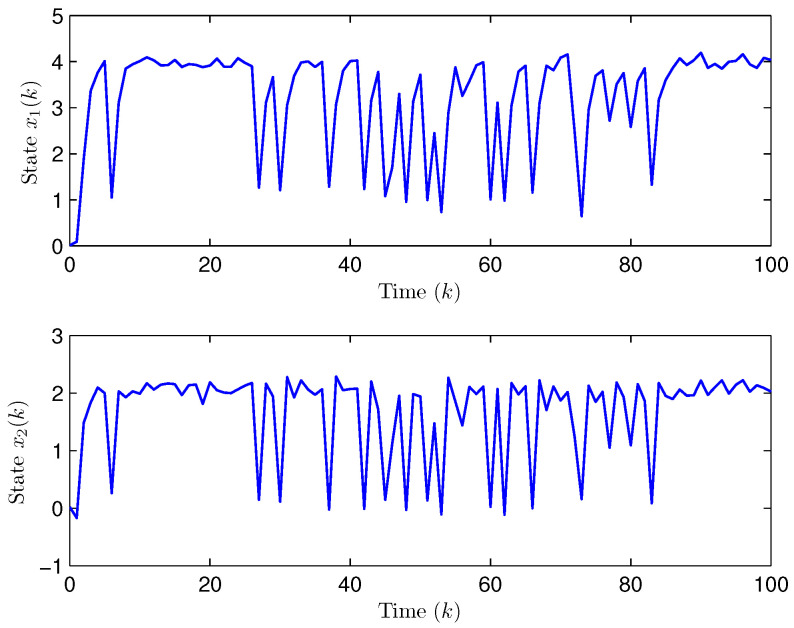
The evolution of state x(k) (Example 1).

**Figure 5:**
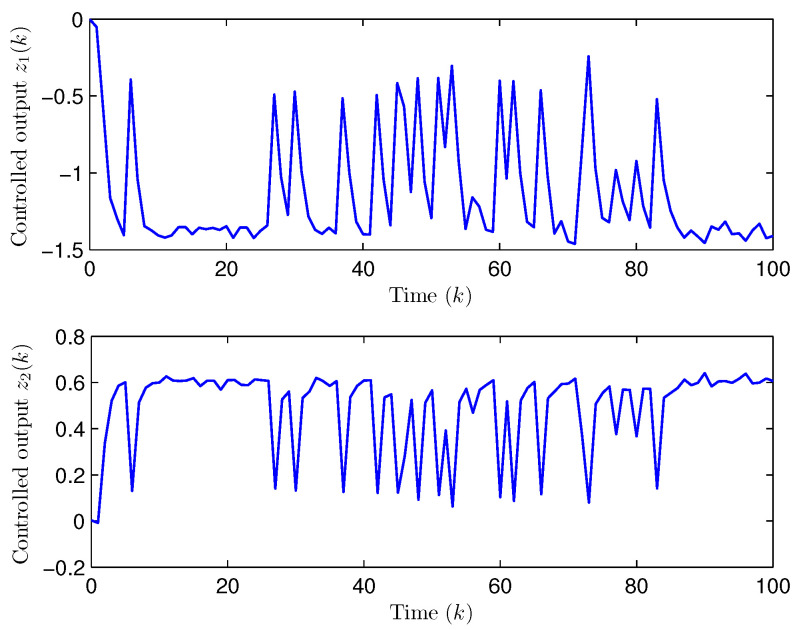
The evolution of controlled output z(k) (Example 1).

**Figure 6:**
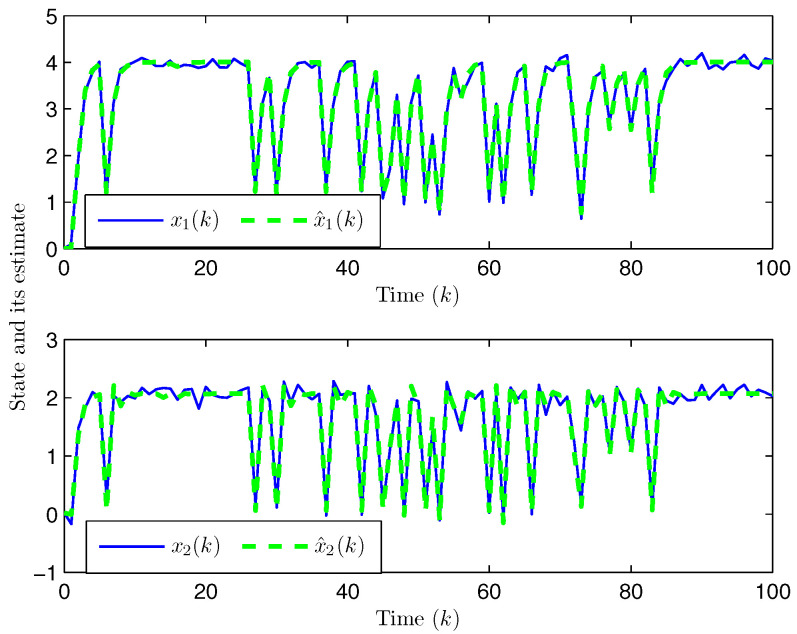
State x(k) and its estimate $\hat{x}\left(k\right)$ (Example 1).

**Figure 7:**
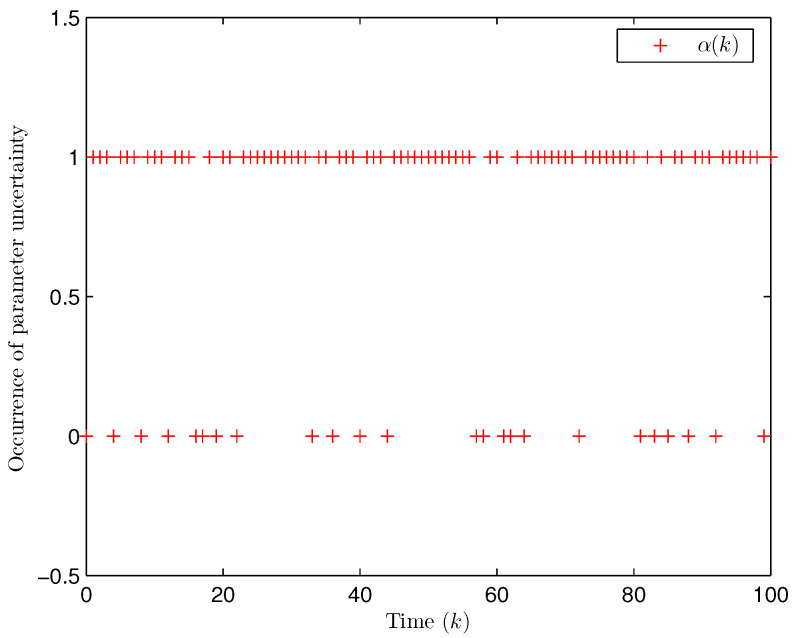
The random occurrence of parameter uncertainty (Example 1).

**Figure 8:**
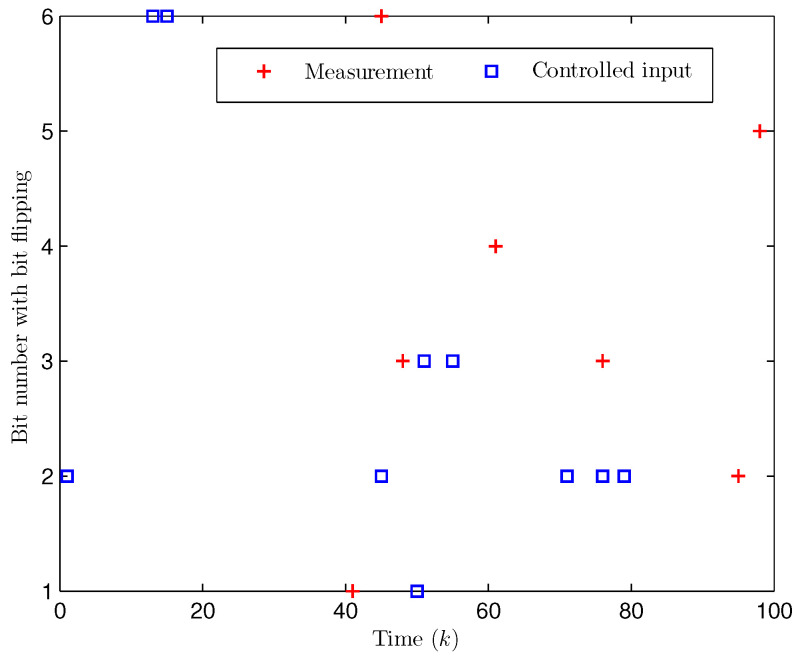
The random occurrence of bit flipping (Example 1).

**Figure 9:**
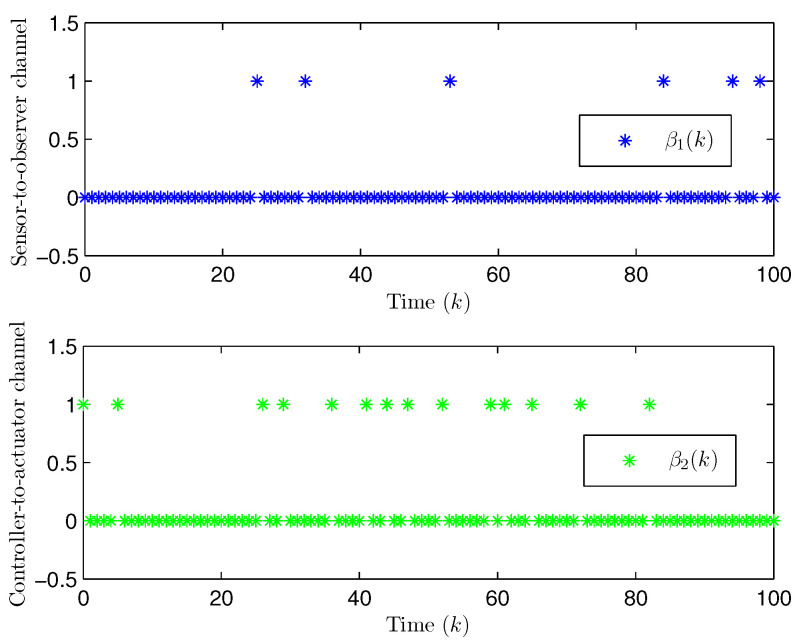
The random occurrence of DoS attacks (Example 1).

*Choose the initial conditions of the system and the observer as $x\left(-2\right)=x\left(-1\right)=x\left(0\right)={\left[\begin{array}{cc} 0.01 & 0.02 \end{array}\right]}^{T}$ and $\hat{x}\left(t\right)={\left[\begin{array}{cccccc} 0.01 & 0.02 & 0.01 & 0.02 & 0.01 & 0.02 \end{array}\right]}^{T}$ (t=−3,−2,−1,0). Set the uncertain matrix as F=diag{sin(2(k−1)),sin(2(k−1))}. Simulation curves are drawn in Figure 2, Figure 3, Figure 4, Figure 5, Figure 6, Figure 7, Figure 8 and Figure 9. Figure 2 presents the evolution of $E\{∥\bar{\eta }\left(k\right){∥}^{2}\}$, which is bounded and within a range [0.0015,62.97]. That is, the closed-loop augmented system (31) meets the EUBMS performance (32). Figure 3 plots the evolution of $E\{∥z\left(k\right){∥}^{2}\}$, which is also bounded and within a range [0.000013,2.532]. That is, the controlled output z(k) achieves EUBMS performance (32). Figure 4 draws the evolution of state x(k) (i.e., $x_{1}\left(k\right)$ and $x_{2}\left(k\right)$, respectively). Figure 5 reveals the evolution of controlled output z(k) (i.e., $z_{1}\left(k\right)$ and $z_{2}\left(k\right)$, respectively). We recognize that both the closed-loop system state and the controlled output are bounded under the designed observer-based PID control method. Figure 6 indicates state x(k) and its estimate $\hat{x}\left(k\right)$, from which we see that the estimate from the observer is accurate. Figure 7 illustrates the random occurrence of parameter uncertainty, we know that when α(k)=1, parameter uncertainty occurs; and when α(k)=0, there is no parameter uncertainty. Figure 8 demonstrates the random occurrence of bit flipping. Taking the control input in Figure 8 as an example, we notice that binary bits at some time steps flip which include (1) for BBS from $\bar{u}\left(k\right)$ at the 50th time step, the 1st bit occurs flipping; (2) for BBSs from $\bar{u}\left(k\right)$ at the 1st, 45th, 71st, 76th and 79th time steps, the corresponding 2nd bit occurs flipping; (3) for BBSs from $\bar{u}\left(k\right)$ at the 51st and 55th time steps, the corresponding 3rd bit occurs flipping; and (4) for BBSs from $\bar{u}\left(k\right)$ at the 13rd and 15th time steps, the corresponding 6th bit occurs flipping. Figure 9 gives the random occurrence situation of DoS attacks, when $\beta_{1}\left(k\right)=1$, DoS attacks occur in the sensor-to-observer channel; and when $\beta_{1}\left(k\right)=0$, DoS attacks do not occur in the sensor-to-observer channel; when $\beta_{2}\left(k\right)=1$, DoS attacks occur in the controller-to-actuator channel; and when $\beta_{2}\left(k\right)=0$, DoS attacks do not occur in the controller-to-actuator channel. Simulation results verify that the designed controller (25) meets the performance (32).*

**Example** **2.** *Now the developed observer-based PID control approach is applied to control the lateral dynamics of an unmanned aerial vehicle (UAV) system with a four-degree-of-freedom model [58,59]. In such an UAV system model ($n_{x}=3$), $x_{1}\left(k\right)$, $x_{2}\left(k\right)$ and $x_{3}\left(k\right)$ stand for the velocity, the track angle, and the track azimuth of the UAV, respectively, and systems parameters are listed below [60]:*

$$\begin{array}{cc} \; & n_{u}=3,n_{z}=3,n_{y}=3,H=0.1I_{\left(3\right)},E=0.5I_{\left(3\right)},C=6I_{\left(3\right)},D={\left[\begin{array}{ccc} 0.02 & 0.02 & 0.02 \end{array}\right]}^{T},\\ \; & A=\left[\begin{array}{ccc} 0.9 & -0.98 & 0\\ 0 & 1 & 0.194\\ 0 & 0 & 1.125 \end{array}\right],B=8\times \left[\begin{array}{ccc} 0.75 & 0 & 0\\ 0 & 0.196 & 0\\ 0 & 0 & 0.196 \end{array}\right],N=\left[\begin{array}{ccc} 1 & 1 & 1\\ 1 & 1 & 1\\ 1 & 1 & 1 \end{array}\right],G=\left[\begin{array}{c} 0.1\\ 0\\ 0 \end{array}\right], \end{array}$$

*and other parameters are the same as those in Example 1.*

*The stochastic nonlinearity function is expressed below:*

$$\begin{array}{c} f\left(k,x\left(k\right)\right)=\left[\begin{array}{c} 0.04\\ 0.05\\ 0.04 \end{array}\right]\times \left(0.2x_{1}\left(k\right)\psi_{1}\left(k\right)+0.3x_{2}\left(k\right)\psi_{2}\left(k\right)+0.4x_{3}\left(k\right)\psi_{3}\left(k\right)\right) \end{array}$$

*where $x_{i}\left(k\right)$ (i=1,2,3) denotes the ith element of x(k), and $\psi_{i}\left(k\right)$ (i=1,2,3) are uncorrelated Gaussian white noise processes with zero mean and unity variance, which are also uncorrelated with w(k) and v(k). We see that such a stochastic nonlinearity fulfills*

$$\begin{array}{cc} \; & E\{f(k,x(k))|x(k)\}=0,\\ \; & E\left\{f\left(k,x\left(k\right)\right)f^{T}\left(k,x\left(k\right)\right)|x\left(k\right)\right\}=\left[\begin{array}{c} 0.04\\ 0.05\\ 0.04 \end{array}\right]{\left[\begin{array}{c} 0.04\\ 0.05\\ 0.04 \end{array}\right]}^{T}E\left\{x^{T}\left(k\right)\left[\begin{array}{ccc} 0.04 & 0 & 0\\ 0 & 0.09 & 0\\ 0 & 0 & 0.16 \end{array}\right]x\left(k\right)\right\}. \end{array}$$

*Solving the optimization issue (57) subject to matrix inequalities (53)–(56), the gain matrices of the observer-based controller are acquired as follows.*

$$\begin{array}{cc} \; & L=\left[\begin{array}{ccc} -10.34200 & -10.34200 & -10.34200\\ -10.50600 & -10.50600 & -10.50600\\ -10.55700 & -10.55700 & -10.55700\\ -0.81915 & -0.81915 & -0.81915\\ -2.55470 & -2.55470 & -2.55470\\ -1.05590 & -1.05590 & -1.05590\\ -0.78285 & -0.78285 & -0.78285\\ -3.51120 & -3.51120 & -3.51120\\ -0.77707 & -0.77707 & -0.77707 \end{array}\right],\\ \; & K_{P}=10^{2}\times {\left[\begin{array}{ccc} 2.67810 & 0.08473 & 0.10941\\ 2.67810 & 0.08473 & 0.10941\\ 2.67810 & 0.08473 & 0.10941\\ 2.67810 & 0.08473 & 0.10941\\ 2.67810 & 0.08473 & 0.10941\\ 2.67810 & 0.08473 & 0.10941\\ 2.67810 & 0.08473 & 0.10941\\ 2.67810 & 0.08473 & 0.10941\\ 2.67810 & 0.08473 & 0.10941 \end{array}\right]}^{T},\\ \; & K_{I}={\left[\begin{array}{ccc} 0.00002 & 0.00002 & 0.00004\\ 0.00128 & -0.00150 & -0.00245\\ -0.00065 & 0.00082 & 0.00134\\ 0.00074 & -0.00138 & -0.00236\\ -0.00011 & -0.00029 & -0.00056\\ 0.00985 & -0.01293 & -0.02134\\ 0.00039 & -0.00353 & -0.00645\\ 0.02895 & -0.03322 & -0.05386\\ -0.00025 & -0.00015 & -0.00034 \end{array}\right]}^{T},\\ \; & K_{D}={\left[\begin{array}{ccc} 0.00327 & -0.00362 & -0.00585\\ -0.00529 & 0.00616 & 0.01001\\ -0.00011 & 0.00038 & 0.00068\\ 0.00573 & -0.00744 & -0.01226\\ 0.00186 & -0.00312 & -0.00529\\ 0.07129 & -0.08051 & -0.13037\\ 0.00977 & -0.01706 & -0.02904\\ 0.20120 & -0.21643 & -0.34793\\ 0.00787 & -0.01142 & -0.01905 \end{array}\right]}^{T}. \end{array}$$

**Figure 10:**
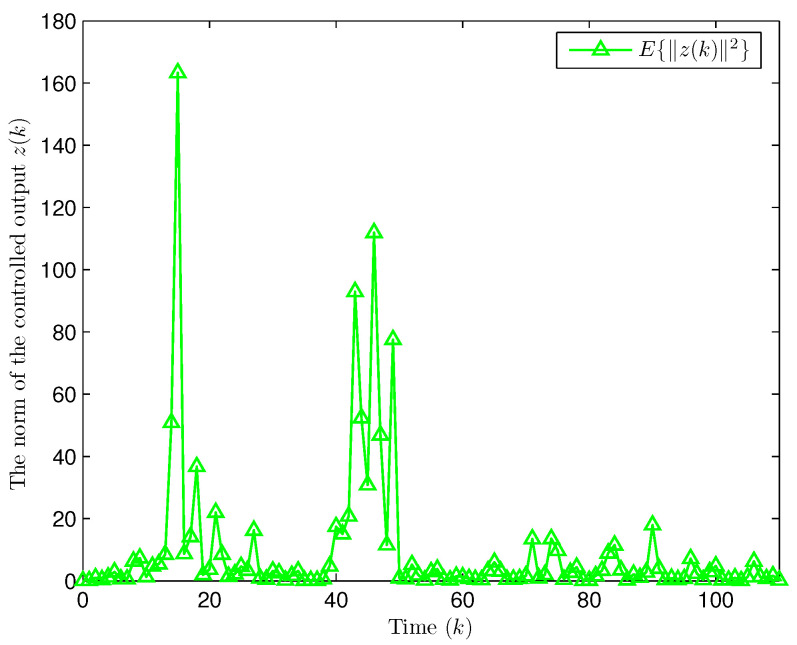
Evolution of the expectation of the norm of z(k) (Example 2).

**Figure 11:**
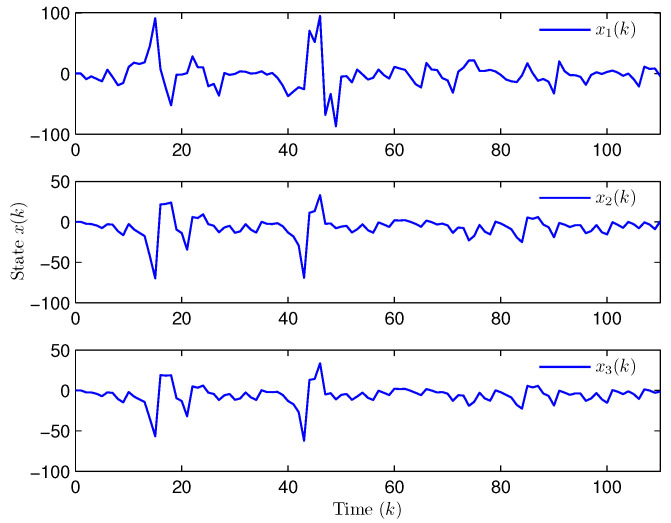
The evolution of state x(k) (Example 2).

**Figure 12:**
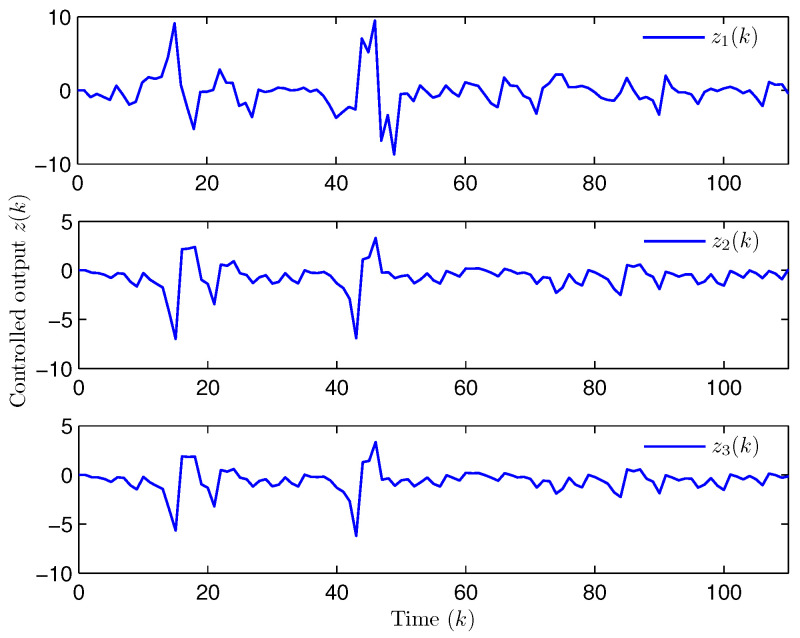
The evolution of controlled output z(k) (Example 2).

**Figure 13:**
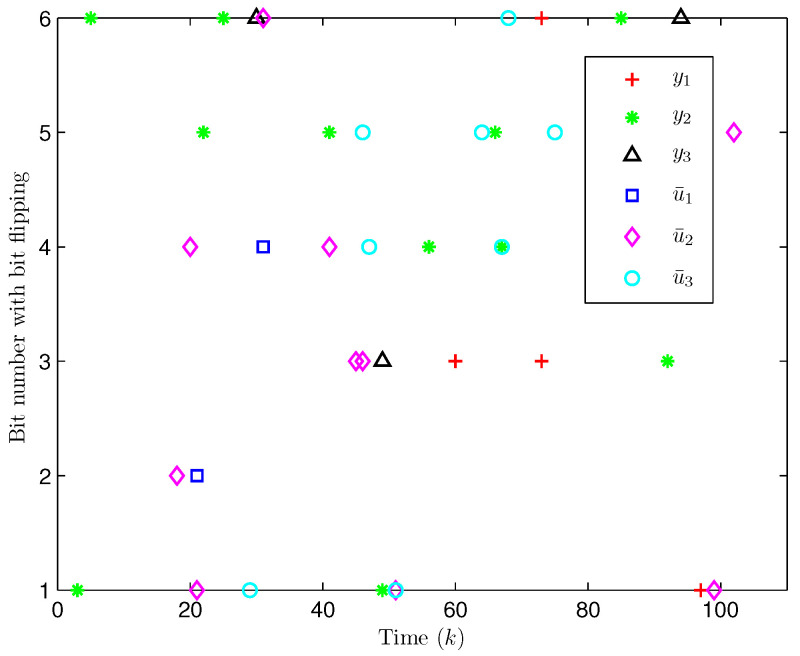
The random occurrence of bit flipping (Example 2).

*Select the initial states of the system and the observer as $x\left(-2\right)=x\left(-1\right)=x\left(0\right)={\left[\begin{array}{ccc} 0.01 & 0.02 & 0.02 \end{array}\right]}^{T}$ and $\hat{x}\left(d\right)={\left[\begin{array}{ccccccccc} 0.01 & 0.02 & 0.01 & 0.02 & 0.01 & 0.02 & 0.02 & 0.01 & 0.02 \end{array}\right]}^{T}$ (d=−3,−2,−1,0). Let the uncertain matrix be F=diag{sin(2(k−1)),sin(2(k−1)),sin(2(k−1))}. Simulation curves are shown in Figure 10, Figure 11, Figure 12 and Figure 13. Figure 10 draws that the evolution of $E\{∥z\left(k\right){∥}^{2}\}$ is within a range [0.000009,163.2986], from which we recognize that z(k) has the EUBMS performance. Under the control functionality of the designed observer-based PID controller (25), Figure 11 and Figure 12 depict the evolutions of the closed-loop system state x(k) and the controlled output z(k), respectively, both of which are bounded. Figure 13 presents the random occurrence of bit flipping during the network channel transmission of $y_{\ell }\left(k\right)$ (ℓ=1,2,3) and ${\bar{u}}_{ℵ}\left(k\right)$ (ℵ=1,2,3) using BES. For example, considering $y_{1}\left(k\right)$ (the 1st component in y(k)) in Figure 13, at the 60th time step, the 3rd bit of BBS from $y_{1}\left(60\right)$ flips; at the 73rd time step, the 3rd bit and the 6th bit of BBS from $y_{1}\left(73\right)$ flip; and at the 97th time step, the 1st bit of BBS from $y_{1}\left(97\right)$ flips. Simulation results point out the correctness of the proposed observer-based PID control approach.*

## 5. Conclusions

This paper has dealt with the observer-based PID control issue for uncertain nonlinear systems subject to integral measurements and DoS attacks using a BES. Integral measurements have been involved to reflect the delayed information acquisition of sensor, which have been coped with via augmenting states at the current and the previous time steps. BES has been employed to transmit the measurement signal through the sensor-to-observer channel and the control input signal through the controller-to-actuator channel, which ensures the quality of data transmission. Random bit flipping has been taken account of to describe the actual transmission situation, and the effect from the resulted bit error has been equivalent to a stochastic noise which promotes subsequent analysis. Randomly occurring DoS attack has been considered to guard against its possible serious influence on the system operation. An observer-based PID controller has been constructed, and the state-feedback control has been realized via estimates from the observer. By virtue of statistical property analysis, Lyapunov stability theory, stochastic analysis skill and matrix inequality techniques, a sufficient condition has been put forth for the existence of the observer-based PID controller, which ensures that the EUBMS performance is achieved of the closed-loop system, and the upper bound is minimized of the ultimate upper bound of the controlled output. Such an observer-based PID controller has been designed whose gain matrices are attained by tackling an optimization issue subject to several matrix inequalities. Two simulation verifications have been done on the developed PID control approach, whose results demonstrate its correctness and usefulness against stochastic nonlinearities, integral measurements, bounded stochastic noises, and randomly occurring uncertainties, bit flippings and DoS attacks. For subsequent research topics, we plan to focus on (1) the security control for multiagent systems [61,62], (2) the security estimation for Markov jump systems [63,64] and dynamic networks [65,66], and (3) the control over relay channels [67,68].

## Data Availability

The original contributions presented in this study are included in the article. Further inquiries can be directed to the corresponding author.

## Abbreviations

The following abbreviations are used in this manuscript:

PID	Proportional-integral-derivative
DoS	Denial-of-service
EUBMS	Exponential ultimate boundedness in mean square
BBSs	Binary bit strings
BES	Binary encoding scheme
BSC	Binary symmetric channel
